# Property Variations of Binder-Free Lignin-Rich Fiber Networks Driven by Forming Processes and Hot Pressing

**DOI:** 10.1021/acsomega.5c06266

**Published:** 2026-01-25

**Authors:** Sara Paunonen, Amanda Mattsson, Gunilla Pettersson, Jukka A. Ketoja

**Affiliations:** † 224809 VTT Technical Research Centre of Finland Ltd, Visiokatu 4, FI-33720 Tampere, Finland; ‡ 6311 Mid Sweden University, Department of Engineering, Mathematics and Science Education, Holmgatan 10, SE-85170 Sundsvall, Sweden; § VTT Technical Research Centre of Finland Ltd, Tekniikantie 21, FI-02150 Espoo, Finland

## Abstract

Sheets made from lignin-rich fiber raw materials can be bonded by hot pressing without external binders. This paper explores how air-laid, foam-laid, and water-laid web formation methods, initial sheet moisture content, as well as hot-pressing conditions (5 MPa, 100–260 °C, 1–60 s), impact the physical properties of board-like materials made of chemi-thermomechanical softwood fibers. In addition to the structural characterization of the hot-pressed materials by X-ray microtomography, air permeance, water contact angle, dry and wet tensile strength, and in-plane compression properties were measured. Despite the significant structural densification, characteristics of the forming method were retained after hot pressing in the final sheet properties. The compressed air-laid sheets had the highest air permeance and the smallest mean pore size, which could be beneficial for particle filtering. At moderate pressing temperatures and times, the significant proportion of large pores in the foam-laid sheets made them weaker than the corresponding water-laid sheets. However, under extreme pressing conditions, the foam- and water-laid sheets reached similar values of high tensile and in-plane compression strength. This suggests that polymer interdiffusion becomes the dominant factor for material strength under these conditions, superimposing the hydrogen bonding created during aqueous forming.

## Graphical abstract

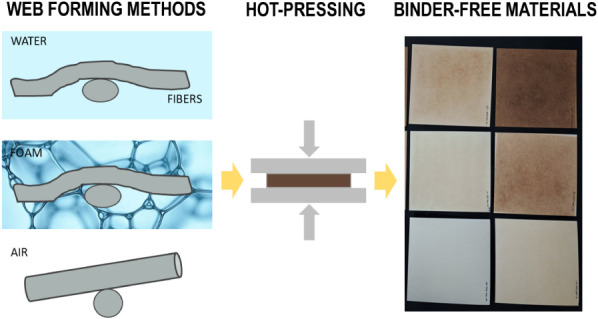

## Introduction

Strength improvement without added binders is an attractive alternative for papers, boards, and cellulosic nonwovens. Eliminating chemical additives would lead to production cost reductions and environmental benefits, as binders commonly contain synthetic or nonbiodegradable components. Hot pressing, a method for improving strength, also allows new properties to be obtained in products, such as water-resistant fiber webs.^1^ Hot pressing is particularly effective for lignin-rich fiber materials, such as chemi-thermomechanical pulp (CTMP) and other so-called high-yield pulps (HYP).^2^ The lignin that naturally resides in fiber walls and surfaces after mechanical pulping^3^ softens when heated, increasing molecular mobility and interdiffusion^4,5^ and, upon cooling, solidifies and improves interfiber bonding. This process has some similarities with the thermal bonding mechanisms of thermoplastic polymers in nonwovens.^6^

As the press temperature increases, hot pressing monotonously improves both the dry and wet^1,5^ tensile strength of HYP fiber sheets, particularly at temperatures well above the glass transition of dry lignin (150 °C). The required lignin content of fibers for significant wet strength improvement by hot pressing is only a few weight percent. There is also an interplay between the press conditions (temperature and pressure) and moisture content (m.c.) of the fibers.^5^ Even though carbohydrates, i.e., hemicelluloses and cellulose, are more strongly affected by moisture than lignin at room temperature,^7,8^ molecular simulations^4^ show a clear reduction in the glass transition temperature and activation energy of lignin with added water molecules. Lignin, a branched and amorphous molecule, softens at about 100 °C when moist and at 150–200 °C when bone-dry,^4,9,10^ depending on its source and isolation method.

Most previous hot-pressing studies have focused on wood and paperboards, both of which are hydrogen bonded lignin-containing materials. Felhofer et al.^11^ demonstrated, using Raman spectroscopy and transmission electron microscopy analyses, that mechanical loading alone can cause lignin migration within the wood cell wall. This tendency is expected to increase at elevated temperatures. Oliaei et al.^12^ suggested that the flow of a lignin-hemicellulose mixture is responsible for the reduced specific surface area in hot-pressed microfibrillated lignocellulose films. However, it remains unclear to what extent lignin transport from the fiber wall contributes to the bonding of initially loose fibers in a dry-formed web. Another open question concerns the relative roles of molecular-level bonding and fiber network geometry in determining the mechanical properties of the structure. This can be investigated by comparing the effects of hot pressing of different types of fiber networks prepared from the same pulp.

Water, foam, and air forming are methods for manufacturing fiber webs. Figure 1 presents their main differences. Water forming, in essence paper manufacturing, involves using low feed concentrations to prevent fiber flocculation and ensure effective fiber distribution. It is the oldest and most widely used production method. Foam forming is a more recent variant where the fibers are transported and laid on wire within an aqueous foam instead of water.^13,14^ Both methods involve water, which softens the fibers and enables hydrogen bond formation and other weak interactions between fibers.^15^ In wet foam, the pulp fibers are confined to the aqueous areas between the air bubbles created by the surfactants and intense mixing, which prevents fiber flocculation and ensures homogeneous webs, even from long fibers. The strength of the resulting material may be affected by surfactant remnants left in the structure after drainage.

**Figure 1:**
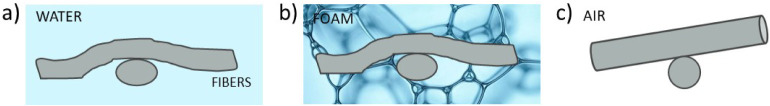
Schematic representation of interfiber contact formation prior to hot pressing. (a) In water forming, the fibers are pliable when there is an abundance of water. As the water drains away, capillary forces draw the fibers together and pack them. Natural fiber bonds are established at contact areas. (b) In foam forming, fiber movement is restricted by bubbles. The fibers are surrounded by water and bonds form between the softened wood fibers. (c) Dry air-laid fibers are rigid and do not bond in the same way.

The third method, air forming or air-laying, involves web formation without water by suspending and transporting dry fibers through an air stream.^16^ In this method, the fibers land randomly on a wire.^17^ In the absence of water, the fibers do not swell, soften, and thus naturally bond. Consequently, air-laid materials require a separate thermal, chemical, or mechanical bonding step. The lack of initial hydrogen bonding caused by web forming usually results in lower mechanical properties of hot-pressed air-laid sheets compared to foam-laid ones.^18^ However, with a significant increase in sheet m.c. (approximately 50%), and adjustments to the pressing temperature and load, air-laid CTMP fiber webs can achieve properties comparable to those of water-laid webs.^19^

The internal structure and surface characteristics of nonwovens, papers, and boards are critical and greatly affect their response to various loads and environmental conditions. For instance, increasing porosity often leads to decreasing mechanical performance. Changes in pore structure can improve air permeability, making the material suitable for applications like filtration. The contact angle, a key property indicating interaction with liquids, is influenced by factors such as chemical composition and surface treatments. Lignin, although it has a total surface energy similar to that of cellulose, is less easily wetted by water due to its higher polar component.^20^

This study explores hot pressing as a bonding method, combining thermal treatment and mechanical compression without adding reactive additives or thermoplastics, and examines the characteristics of hot-pressed fiber networks. The focus was on dense (700 kg/m^3^ to 1100 kg/m^3^) and relatively heavy (450 g/m^2^) paperboard-type materials. The goal was to study the extent to which structural variations in the sheet arising from the forming process influence the properties of the final sheet after hot pressing. Foam-laid sheets have a significant proportion of large pores^21^ that tend to merge and form air channels through the sheet.^22^ In contrast, water-laid sheets usually lack such channels^18^ but are less homogeneous on a larger scale.^14^

This work increases knowledge related especially to the waterless air-laid forming method. Pore structure in air-laid sheets is influenced by the type and amount of bonding agent and the overall fiber distribution. Therefore, a detailed comparison of the forming methods is meaningful only after they have been similarly bonded and have roughly equal density. To our knowledge, this study provides such a comparison for the first time. The results show that the initial fiber organization and bonding is reflected in the sheet structure after hot pressing, resulting in different property ranges for each production method.

## Materials and Methods

### Materials

Bleached chemi-thermomechanical pulp (CTMP) from softwood, with a Canadian standard freeness (CSF) of (560 ± 40) ml (approximately 18°SR), was used as dry fluff for preparing the air-laid, foam-laid, and water-laid samples. To foam the fiber-water mixture, a surfactant mixture (1:1 by weight) of sodium dodecyl sulfate (SDS, purity at least 90%) and polyethylene glycol sorbitan monolaurate (Tween 20), both from Sigma-Aldrich (Germany), was prepared.

### Fiber Properties

The chemical composition of the raw material CTMP fibers (solids content 91.3%) was determined using the process described in the Supporting Information. The total lignin content was 26.8%. This is at the same level as is typically reported for unbleached softwood CTMP fibers (from 20% to 30%). According to the calculations, the total carbohydrate content (70.9%) was divided into 41.4% cellulose and 31.7% hemicellulose. There were less than 0.1% of extractives. The CTMP fiber dimensions were measured by L&W Fiber Tester Plus (Lorenzen and Wettre, Sweden) according to ref 23 and are shown in Table 1.

**Table 1:** Geometrical Properties of the Dry CTMP Fibers^b^

Mean fiber length (mm)	Mean fiber width (μm)	Aspect ratio (−)	Mean fines^a^ (%)
1.63 ± 0.08	36.8 ± 0.6	44.2	65.9 ± 0.8

a Length-weighted share of particles with a size of less than 0.2 mm.

b The values represent the average of two repeat samplings.

### Air-Laid Web

A continuous, unbonded air-laid CTMP web (width 40 cm) with an approximate grammage of 450 g/m^2^ was prepared with a Dan-Web pilot machine at UPM (Rauma, Finland). The fibers were extracted from a bale with a chisel, fed into a hammermill that separated them, and transported in an air stream to the forming drum, after which the web was collected in loose rolls of 2 m each. The air-laid (AL) samples studied were obtained by directly cutting them from these rolls. In addition, the air-laid web (Figure 2) also provided the raw material for preparing the foam-laid and wet-laid samples.

**Figure 2:**
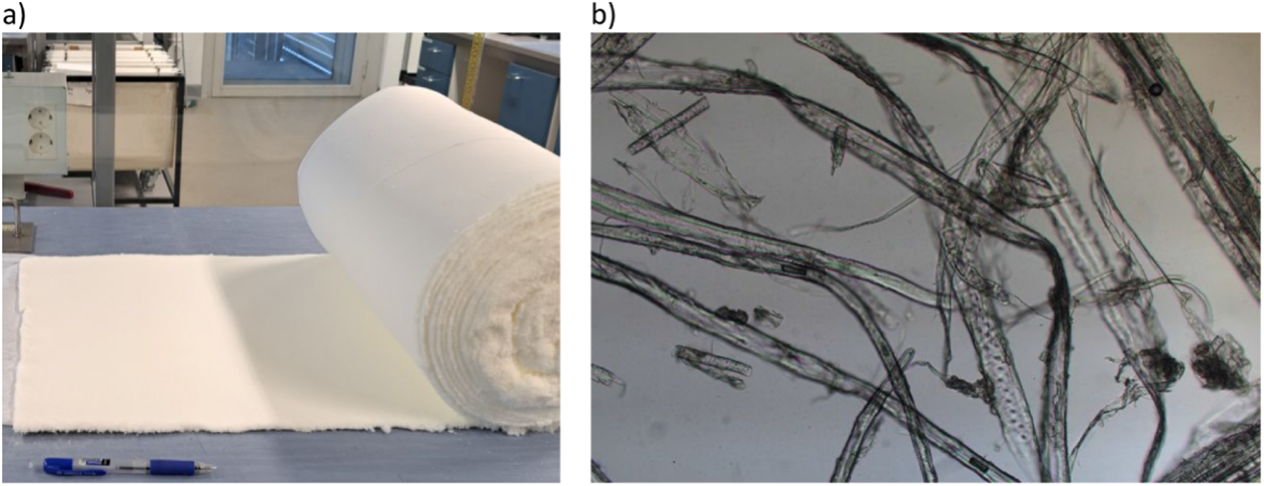
(a) CTMP air-laid web with a width of 40 cm, and (b) light microscopy images of CTMP fibers.

### Preparation of Foam-Laid and Water-Laid Sheets

The target grammage of the foam- and water-formed samples was the same as for the air-laid web, 450 g/m^2^. First, pieces of CTMP air-laid web were wet disintegrated at a consistency of 4%. To prepare the foam-laid (FL) sheets, pulp (consistency 2%) and surfactant (0.6 g/L) were mixed in a cylindrical foaming vessel. The initial volume of the suspension was 2 l. By mixing with a bent disc-type mixing plate (Ø 83 mm) in a Netzsch Shearmaster mixer (NETZSCH Grinding and Dispersing, Germany), the volume was increased to 6 l and the air content to 66%. The foam was poured into a hand sheet mold along a tilted plate. Excess water was removed from the sheets with a vacuum. The sheets were wet pressed between blotting paper and a metal plate at a pressure of 0.34 MPa for 7 min and subsequently dried at 105 °C in a KRK Rotary Dryer DR-200 (Kumagai Riki Kogyo, Japan) that largely prevented sheet shrinkage.

At the end of the foaming stage, the bubble size distribution was determined by image analysis using a method described in ref 24. A small amount of aqueous foam was drawn into a cuvette with 1.6 mm spacing between two glass plates. Two parallel images of the bubble structure were recorded within 30 s using an optical microscope. Bubbles were automatically detected after image thresholding using the Circular Hough Transform. The area-weighted (Sauter mean) diameter of bubbles was found to be 91.6 μm, 89.6 μm, and 87.4 μm in three tests.

The water-laid (WL) samples were prepared from air-laid web pieces as standard hand sheets, according to ISO 5269-1.^25^ Disintegration was carried out at a consistency of 40 g/L. Water was removed by wet pressing, as with the foam-laid samples, followed by drying with the KRK dryer.

### Hot Pressing

For hot pressing, the sheets were conditioned at 50% and 90% relative humidity (RH) to moisture contents (m.c.) labeled as “Dry” and “Moist”, according to Table 2. At both RH levels, the m.c. of the AL sheets was about 1.5% points higher than the m.c. of the FL and WL sheets. In contrast to the AL sheets, the FL and WL sheets underwent a cycle of wetting, disintegration in water, and drying.

**Table 2:** Initial Moisture Contents of Air-Laid (AL), Foam-Laid (FL), and Water-Laid (WL) Sheets^a^

		Initial sheet moisture content [%]^a^		
Label	Conditioning	AL	FL	WL
Dry	23 °C, 50% RH, > 24h	10.3 ± 0.5	8.7 ± 0.5	8.3 ± 0.5
Moist	23 °C, 90% RH, > 24h	18.4 ± 0.5	16.9 ± 0.5	16.8 ± 0.5

a Percentage of oven dried weight (105 °C).

Hot pressing was performed on the pressing machine shown in Figure 3, containing heating blocks and pillar stand. This setup was integrated into a servo-hydraulic testing system (MTS Systems, USA) and controlled by the software program FlexTest60 and Multi-Purpose Test ware (MPT), both from (MTS Systems, USA).^26^ The sample between perforated papers was placed in the hot-press with a small 20 mm plate gap, after which the hot bottom plate moved rapidly up toward the fixed upper one.

**Figure 3:**
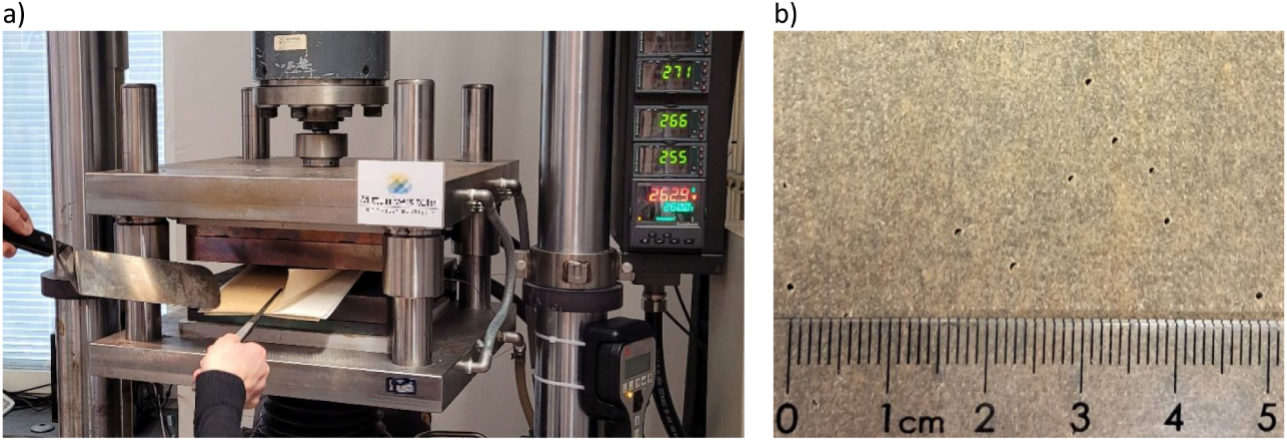
(a) The hot-pressing equipment where the metal plates on both sides of a sample are heated to equal temperature. (b) The perforated paper surrounding the sample has small holes (0.15 mm) for general use and additional larger holes (0.8 mm) for pressing moist sheets, to aid steam control during hot pressing.

The applied pressure was 5 MPa (100 kN of force on 142 mm × 142 mm plates) for the time given in Table 3, followed by a three-second after-hold at 0.17 MPa (force of 3.5 kN). The value of 5 MPa was selected because it lies within the pressure range commonly reported for laboratory-scale static (3.5 MPa),^4^ and continuous (7 MPa),^1^ press studies, as well as continuous industrial processes (5 MPa).^27^. It also corresponds to pressure levels typically used for molded pulp articles (1–6 MPa),^28^ and 3D forming of air-laid materials (4–40 MPa),^29^. The after-hold allowed any remaining moisture to evaporate from the sheet without causing dimensional changes in the sample. The press plate temperatures are also shown in Table 3. To prevent steam explosions, the samples were enclosed in densely perforated (hole diameter 0.15 mm) baking paper (see Figure 3) with blotting paper at the bottom. For the moist sheets, the baking paper was also supplemented with slightly larger (hole diameter 0.8 mm) holes made with a thin needle in a random pattern.

**Table 3:** Hot-Pressing Conditions for AL, FL, and WL Samples and References^a^

Label	Plate temperature [°C]	Hot-pressing time [s]
*Samples:*		
Dry	220	1, 10(*), 60
	260	1, 10(**), 60
Moist	220	1, 10, 60
	260	1, 10(*), 60
*References:*		
Dry	100	10(*)
Dry	–	Unpressed FL and WL

a The applied pressure was 5 MPa, followed by an after-hold stage of 0.17 MPa for 3 s. (*) Samples from which CT-scans (two repeats) were taken. (**) CT-scans were taken only from the AL sample.

### Sheet Testing

Prior to testing, all samples were conditioned at (23 ± 1) °C and (50 ± 2) % RH according to ISO 187.^30^ The standard sheet thickness and density were measured according to ISO 534:2011,^31^ and the grammage according to ISO 536:2019.^32^ Air permeance was measured using an L&W air permeance tester (ABB/Lorentzen and Wettre, Stockholm, Sweden) with the Bendtsen method, in accordance with ISO 5636.^33^

The dry tensile strength properties were determined using a Zwick Roell Z2.5 TN tensile tester (Zwick, Ulm, Germany) equipped with an Xforce load cell (2.5 kN), and standard specimens (15 mm width) according to ISO 1924-3:2005 ^34^ (test speed 100%/min). The wet tensile strength was measured according to ISO 1924-3:2005 ^34^ (same test speed) after immersion in water for 1 min. ALs and FLs were tested for machine direction (MD) and cross-machine direction (CD) (five test specimens in each direction), whereas WLs were assumed isotropic (10 specimens). After hot pressing, the orientation anisotropy based on the average ratio of tensile stiffness in MD and CD was 1.09 for ALs and 1.20 for FLs. In other words, these samples were almost isotropic as well. To remove any effect coming from anisotropy, the geometric average of the dry and wet tensile strength indices (Dry-TI, Wet-TI) and their propagated errors are reported. For WLs, the average TI and its standard deviation are reported. A short-span compression test (SCT) with a 0.7 mm free span was performed at a test speed of 3 mm/min using an L&W SCT tester (ABB/Lorentzen and Wettre, Stockholm, Sweden) according to ISO 9895:2008.^35^

To verify hydrophobicity, static water contact angles (WCAs) were measured using a PGX+ Pocket Goniometer (PTE, Sweden). A 4 μL water droplet was placed on the surface and the contact angle was recorded from the images taken until the droplet was fully absorbed. The reported values represent the average of three measurements.

### SEM Microscopy

To investigate the surface morphology, images were acquired using a high-resolution Tescan Maya3–2016 scanning electron microscope (SEM) (TESCAN Brno, s.r.o., Brno, Czechia). Prior to imaging, the samples were sputtered with a thin layer of 5 nm iridium. The electron beam voltage was set to 4.00 kV and the beam intensity was 1.00. A secondary electron detector was used to capture images at a working distance of 6 mm to 7 mm from the samples.

### Optical Formation

Sample formation was measured optically using transillumination and digital imaging. In VTT’s system, the sample is placed between an opal and a clear glass plate to eliminate variation in distance from the light source. The sample is backlit with a Schott Fostec DCR III light source (Schott Optics, USA). The light reaching the PCO SensiCam CCD camera (Excelitas, USA) is kept constant by adjusting exposure time. The image area is 74 mm × 59 mm.

### X-ray Microtomography

For pore structure analysis and 3D visualization, microcomputed X-ray tomography (XμCT) images were taken of selected samples using a desktop microtomography scanner (RX Solutions, France). The imaging, based on X-ray attenuation maps obtained from multiple positions around the sample, captures material density differences in the sample. Two XμCT scans were performed on the materials listed in Table 3. The image area was 6 mm × 6 mm (pixel size 4.0 μm). Thresholding was applied to the images to create a segmentation of the fibers, as described in ref 36. The porosity and pore size probability density (PD) function were reported. Pore size PD was obtained by fitting the maximum void diameter to each voxel. The number of voxels was then plotted against the normalized diameter volumes relative to the total sample volume.

## Results and Discussion

### Sheet Surface Properties

All sheets experienced browning as the hot-pressing temperature and time increased, as shown in Figure 4. The degradation of lignin has been shown to occur within the temperature range of 200–500 °C, while for hemicellulose, it occurs between 230 and 315 °C, which explains this color change.^9,37^ Darker spots are observed in the ALs, where the intensity of the hot pressing correlates with the darkness of the knots, as shown in Figure 4. Fiber individuation with a hammermill does not usually fully separate the dry fibers, leaving some parts as knots (fiber flocs), which is considered a quality criterion of fluff pulp. From a tactile perspective, the ALs felt more pliable, bendable, tougher, and more resistant to sharp folds and cracking.

**Figure 4:**
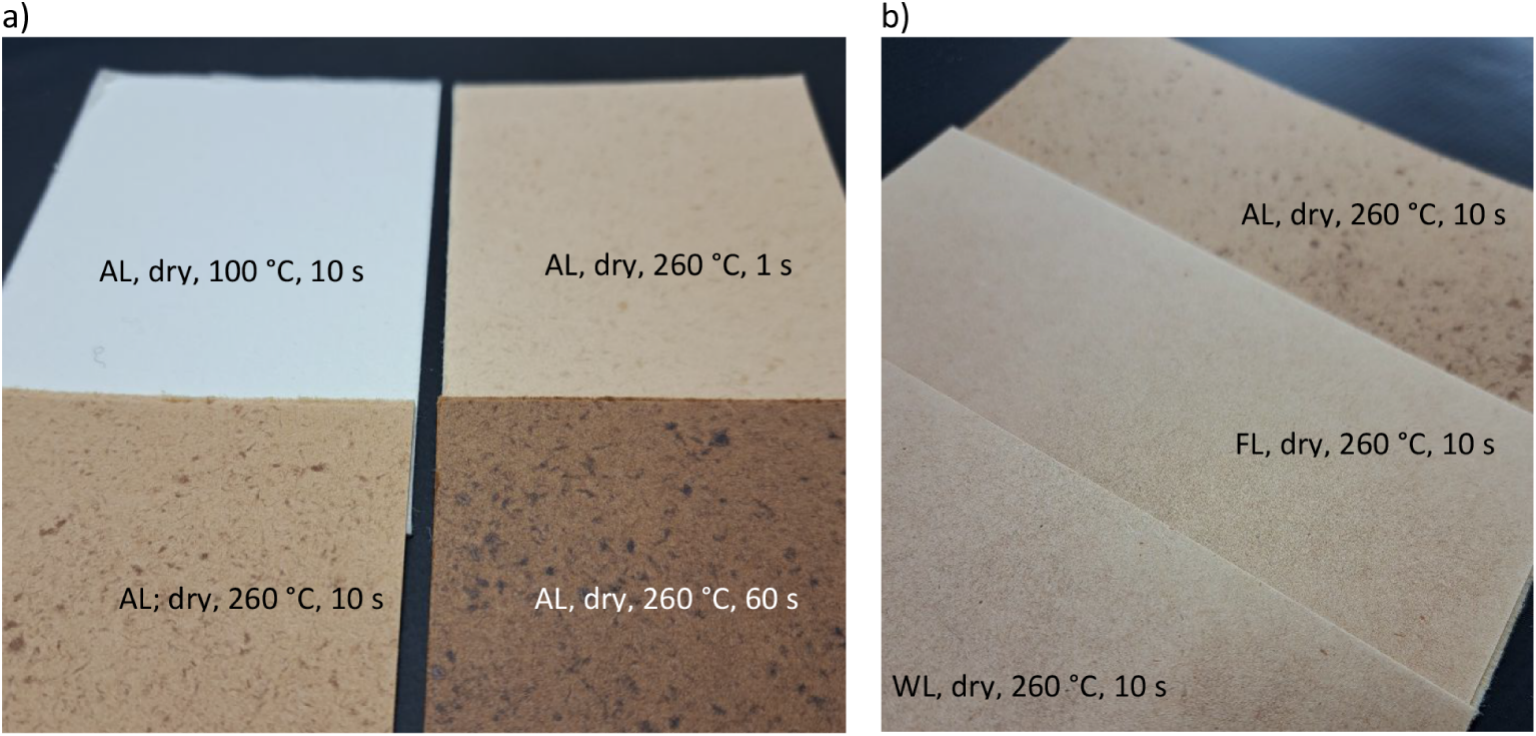
Photographs of sheet surfaces highlighting the browning behavior under harsher conditions and differences in formation. (a) AL samples hot-pressed for increasing times. (b) Three different forming methods hot-pressed at 260 °C for 10 s (right). Sheet size 14 cm × 14 cm.

The SEM images in Figure 5 illustrate the sheet surface morphology of the AL, FL, and WL samples. The upper row shows sheets pressed dry at 100 °C for 10 s, a reference point to establish a baseline. In these conditions, the fiber networks were relatively open and porous. The fibers in the ALs appeared more flexible, whereas the FL and WL fibers had a stiffer and more rigid appearance. This suggests that fiber aggregation was more pronounced for FL and WL samples, resulting in wider pore-size distributions at a certain average material density. A detailed discussion is provided in the section Internal Sheet Structure Characteristics.

**Figure 5:**
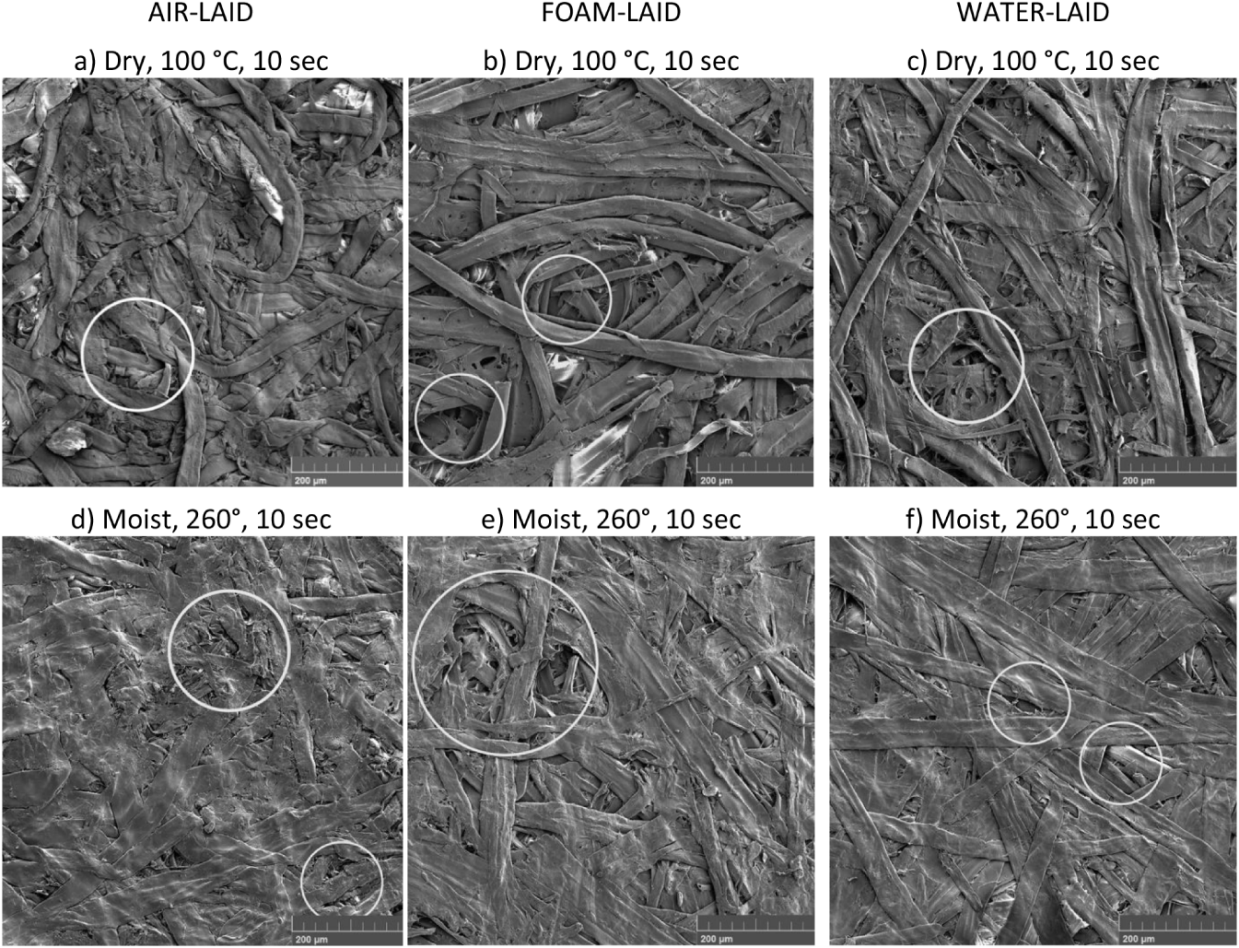
SEM images of AL, FL, and WL sheet surfaces under (a–c) mild (Dry, 100 °C, 10 s) and (d–f) intense (Moist, 260 °C, 10 s) hot-pressing conditions. Magnification 200×. The images highlight the smoothing and flattening effects of intense hot pressing, with some surface pores visible within the circled areas. In the (d) ALs, these pores appear smaller than in the (e) FLs or (f) WLs, which is consistent with the X-ray tomographic structural analysis discussed later.

The lower row displays sheets pressed moist at 260 °C for 10 s. Compared to the upper row, these conditions clearly led to significant densification of the structures. The consolidation of the sheets differed slightly depending on the method, with the surface pores in the AL sheets being smaller compared to the FL and WL sheets. Additionally, the fiber boundaries in the AL samples were less pronounced, suggesting that the lignin has softened more easily above its *T*_g_ and has been redistributed on the surface, likely due to the higher m.c. of the AL fibers (Table 2). It has been proposed that this distribution or interdiffusion of lignin molecules^5^ enhances fiber–fiber bonding, potentially leading to changes in the surface properties.

Water contact angle (WCA) measurements provide a quantitative assessment of a solid surface’s wettability by water. Table 4 summarizes the results for the sheets on which a water droplet remained on the surface for over 20 s. The highest WCA values were obtained for samples pressed at 260 °C or with increased moisture content. Hot-pressed AL samples generally exhibited higher WCA values than the FLs and WLs. Under milder conditions (lower pressing temperature or lower sheet m.c.), AL surfaces showed slow enough liquid absorption to allow measurement of a contact angle after 20 and 40 s. In contrast, under similar conditions, no measurable WCA was obtained for FLs or WLs; therefore, these results are not presented in Table 4.

**Table 4:** Water Contact Angle, Measured on the Sheets after 20 s and 40 s^a^

Forming method	Initial m.c.	Temperature [°C]	Hot-pressing time [s]	WCA [°] after 20 s	WCA [°] after 40 s
AL	Dry	260	60	67 ± 4	51 ± 5
AL	Moist	220	60	66 ± 9	61 ± 14, n=2
AL	Moist	260	10	69 ± 5	65 ± 8
AL	Moist	260	60	78 ± 7	75 ± 5
FL	Moist	260	10	56 ± 4	48 ± 0, n=2
FL	Moist	260	60	52 ± 4	49 ± 6
WL	Moist	260	10	58 ± 3	52 ± 4
WL	Moist	260	60	53 ± 14	46 ± 26, n=2

a Average ± SD, *N* = 3. All measurements were performed as triplicates (*N* = 3). For some specimens, absorption was too fast to get a result for the material (*N* = 2).

In fluff pulp manufacturing, chemicals are often added to the pulp prior to drying to promote fiber separation during mechanical hammer milling into low-density fluff. These debonding agents are typically surfactants that adsorb onto fiber surfaces in water and partially inhibit the natural hydrogen bonding between fibers.^38^ In the dry state, the fibers retained these surfactants, which likely influenced the measured surface properties of the ALs. However, when the fluff pulp was rewetted to prepare the FL and WL sheets, the chemicals could have leached out. This difference in surface chemistry together with well-spread lignin may explain the higher WCA values detected for the ALs.

In addition, despite their lower density (Figure 7) and strength (Figure 11), the ALs also showed a more evenly consolidated sheet surface with smaller pores, potentially also contributing to reduced wettability and higher WCA values. This is evident also in the cross-sectional CT images (Figure 9). The highest average WCA value was 78° ± 7° after 20 s, with an initial contact angle well above 90° (Figure 6) which is considered hydrophobic surface.^39^ Over time (see Table 4), the WCA decreased due to surface fiber wetting, until the droplet was fully absorbed within 2–3 min.

**Figure 6:**
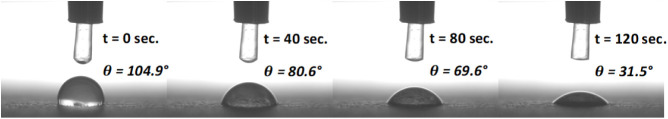
Contact angle immediately after applying the droplet to the AL sample, moist, 260 °C, 60 s.

### Internal Sheet Structure Characteristics

Hot pressing caused a dramatic densification of the sheets studied (Figure 7). This effect increased with elevated temperature, moisture, and pressing time. The FL and WL samples had been wet pressed at room temperature prior to drying, leading to densities of (312 ± 10) kg/m^3^ (FL) and (372 ± 4) kg/m^3^ (WL) for these initial sheets. Still, hot pressing roughly doubled (at 100 °C) or tripled (at 220 and 260 °C) their density. As to the polymer components of wood fibers, the softening temperature of dry lignin is about 150 °C, but this temperature is reduced with increasing moisture content of lignin.^40^ Moreover, moisture also softens hemicelluloses and increases the separation of crystal microfibrils,^41^ which makes pulp fibers more pliable during compression. In addition to the mechanical pressure applied, all the above factors contributed to the collapse of the fiber lumens (Figures 5d–f, and Supporting Information, Figures S9 and S10) and the large interfiber pores with diameters of 60–80 μm (Figure 8). Even so, smaller pores were left in the structure (Figure 8) and microsized gaps between the fibers could have remained in the fiber joint regions.

**Figure 7:**
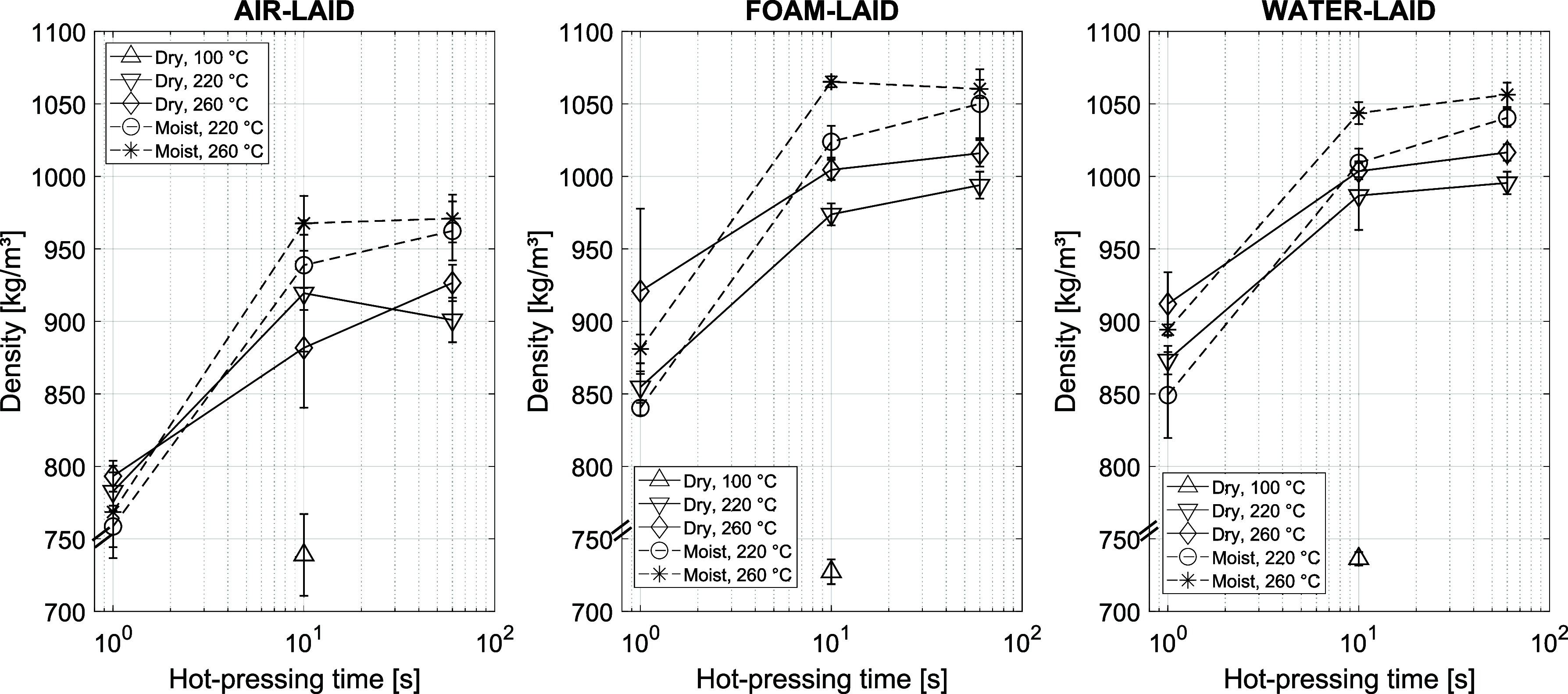
Sheet densities after hot pressing. Unpressed references: AL 50–100 kg/m^3^ (5 mm thick mat), FL (312 ± 10) kg/m^3^, WL (372 ± 4) kg/m^3^. Unpressed, nonbonded AL is a loose material without a well-defined thickness.

**Figure 8:**
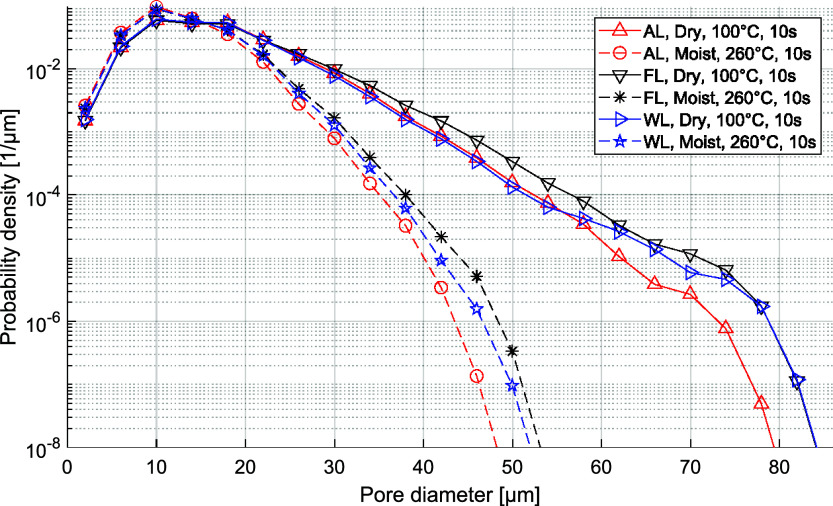
Probability density of different pore diameters for AL, FL, and WL sheets under mild (Dry, 100 °C) and severe (Moist, 260 °C) hot-pressing conditions. Data averaged from two X-ray microtomography scans per condition. The average porosity was almost equal for the different forming methods under the same pressing conditions (see Supporting Information, Figure S3 for porosity data). Because of the logarithmic *y*-axis, the differences in pore-size distributions between the forming methods are larger than they may at first appear.

Heat exchange and thermal diffusivity in cellulose fiber materials have been studied extensively due to their importance in papermaking, converting, and end use of paper.^42^ The current hot-pressing conditions are similar to those in high intensity (impulse) drying, where the heat transfer coefficient increases with applied pressure. At a pressure of 3.5 MPa (lower than in this study), the estimated coefficient is already approximately 3500 W/(m^2^K).^43^ The reported thermal diffusivity values of cellulose fiber networks are on the order of 2 × 10^−7^ m^2^/s across a broad range of paper grades and densities (360–1100 kg/m^3^).^44^ Based on earlier temperature data on hot pressing of thick paper,^43,45^ the dry samples were expected to reach the softening temperature of lignin in less than 1 s after contact with the hot-pressing plates.

However, the density changes in all samples between 1 and 10 s of pressing were larger than that obtained by a temperature variation of 220 to 260 °C or by added moisture. This suggests that structural deformations during compression from 1–10 s were time-dependent and thus viscoelastic^46^ and could be speeded up and enhanced by temperature and moisture, as seen in Figure 7. In general, a similar acceleration effect could probably be observed with increasing pressure, as earlier creep studies have shown that the rate of viscoelastic changes in lignocellulosic fiber networks increases nonlinearly with applied stress.^46,47^ With a longer hot-pressing time, the main structural deformations affecting density appeared to be saturated, but polymer-level changes affecting, e.g., strength properties could have continued.

The sheet forming method affects the structure on at least two different scales. First, the μm-scale pore diameter distributions for different sheet types deviate at the low 100 °C pressing temperature (Figure 8), even when the sheet density is the same in all cases (Figure 7). The collapsed pores are very flat, and in planar directions the pore size differences are expected to be much larger than what is seen in Figure 8. These characteristic differences are inherited in the sheet structures even after the most severe pressing conditions of 260 °C for moist sheets, as shown in Figure 8. The foam-formed sheets had the highest proportion of large pores, created as traces of aqueous bubbles during sheet forming. In contrast, the pores have the smallest mean size in the AL sheets for both mild (Dry 100 °C) and severe (Moist 260 °C) compression conditions. Thus, this feature is not due to differences in fiber softening during pressing, but rather from the characteristic microscale network structure. The microporous structure of WL sheets lies between that of ALs and FLs.

The second structural effect derived from the forming method is seen in the so-called “formation” (millimeter) scale, which is related to the distribution of fibers over the plane directions during forming. Due to the inherent variation in the AL forming process, the AL base webs had a larger millimeter-scale grammage variation than the FL or WL samples. In air forming, fiber deposition is less controlled than in foam or water forming because of the low air viscosity. In contrast, the FL and WL sheets were prepared one by one using water or foam as a transfer medium, which helped to distribute the fibers evenly over a forming fabric. The average grammage was quite similar for all three sample types (Supporting Information, Figure S1), and any small differences between these values for the different forming methods were not significant. However, the grammage variation within a single sheet played a more important role, as it affected the local pressure during compression and, therefore, the planar porosity variation of the sheet. This can be seen in Figure 9 for the three sample types.

**Figure 9:**
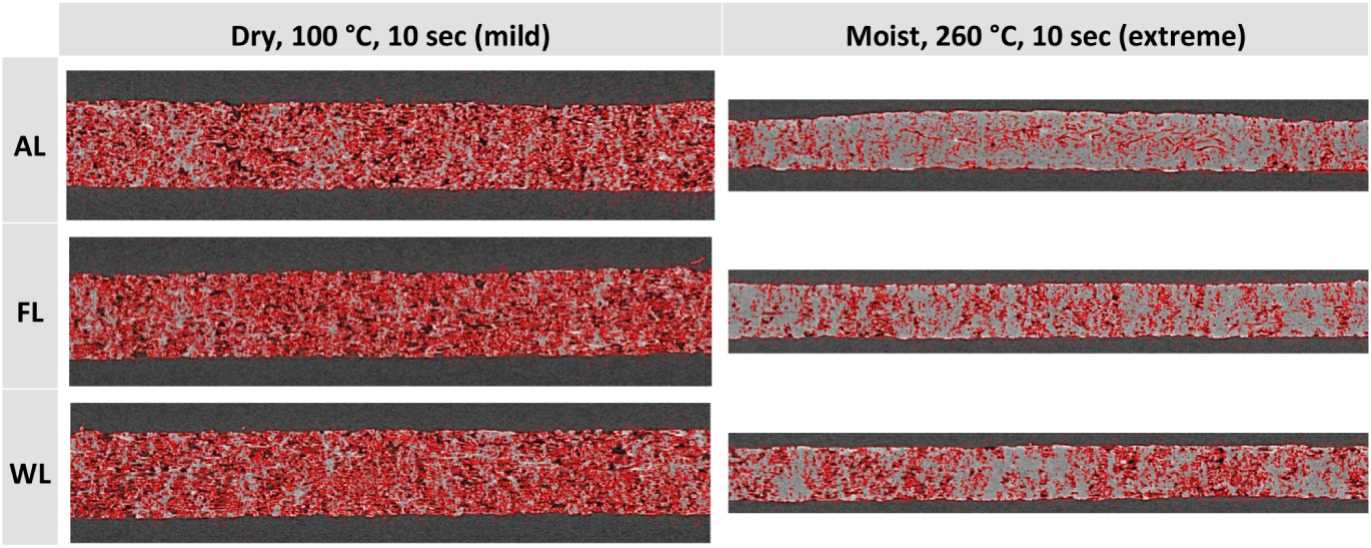
Cross-sectional illustrations from CT scans showing sheet densification by hot pressing in mild and extreme conditions. The boundaries between the fiber material and the void spaces are shown in red. The sample width is 5.7 mm in all cases.

In the samples hot-pressed at 260 °C (Figure 9), one can see high-density regions where the visible gaps (shown in red) between the fibers disappear. On the other hand, there are also regions with a high number of pore interfaces extending throughout the sheet thickness. According to the optical formation measurement (Table 5), which shows the surface structure of the high-density samples, the AL sheets were the least homogeneous at the millimeter scale. This can be seen in their highest gray scale variation (Stdev) and largest mean floc size (Floc size). With greater grammage variation, the average sheet density of the hot-pressed AL sheets was lower than that of the corresponding FL and WL sheets (Figure 7) when pressed above the lignin softening temperature. However, densities ((730 ± 30) g/m^3^) and thicknesses ((626 ± 2) μm, Supporting Information, Figure S2) were nearly the same in all cases when pressed at 100 °C for 10 s (Figure 9), and inner porosity (void fraction) was almost the same (Supporting Information, Figure S3) for all forming methods after pressing at even higher temperatures. One should also note that millimeter-scale homogeneity was excellent for the FL sheets (Table 5), even though they had larger pores after compression (Figure 8). This shows how the level of structural heterogeneity can be scale-sensitive and thus affect the properties of the product under study in different ways.

**Table 5:** Optical Formation of Hot-Pressed Samples under Mild Conditions (Dry, 100 °C, 10 s)^a^

ID	Mean	Stdev	Std/35 mm	Floc size [mm]
AL, mild	2083	197	140.63	1.30
FL, mild	2054	90	71.60	1.00
WL, mild	2072	107	85.67	0.86

a Mean and standard deviation of grayscale values in the image (Mean, Stdev). Standard deviation after applying a 35 mm filter (Stdev/35 mm). Calculated floc size (floc size ). Average of two Images

Differences in local grammage, density variations, and pore structure between different hot-pressed sheet types are reflected in the measured air permeance (Figure 10), which is a fundamental property for several end uses. The longer the hot-pressing time, the smaller the differences between the forming methods. The combined effect of initial m.c. and pressing time was most evident in the ALs. At 10 s, the permeance of moist AL sheets had almost reached its minimum, whereas the densification of dry sheets continued, which was seen in air permeance as well.

**Figure 10:**
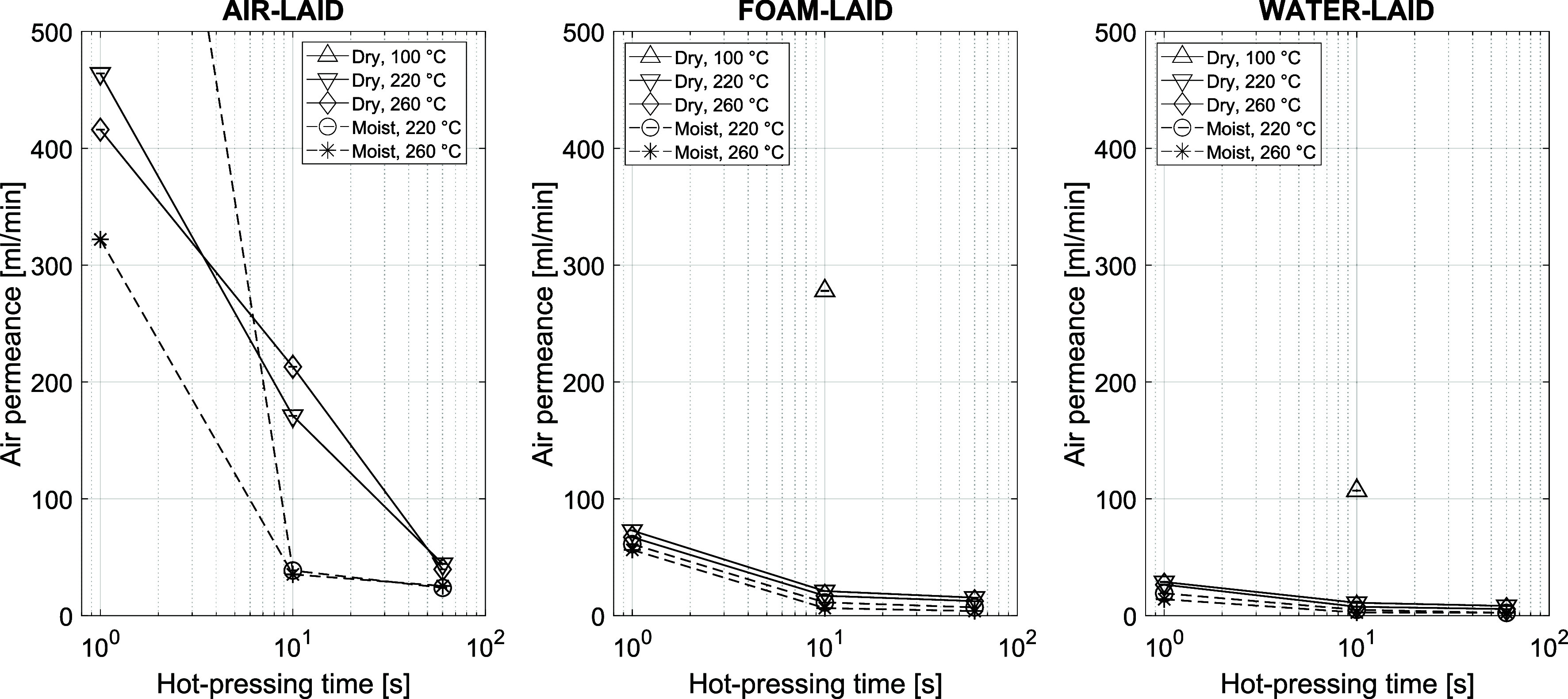
Air permeance. The data point for AL (Moist, 220 °C, 1 s), 1090 mL/min, is outside the graph. References (100 °C, 10 s): AL 1160 mL/min (outside graph), FL 278 mL/min, WL 107 mL/min. Unpressed references: FL 7820 mL/min, WL 1070 mL/min. A single data point is reported for each material; error estimation is not available.

Previous studies have shown that FL sheets have a higher air permeability than WL sheets at equal density.^48^ According to a previous model analysis,^49^ the reason for this is that the large pores in a foam-formed fiber network create more direct flow channels. This higher air permeance of FLs compared to WLs was seen also in Figure 10 after the hot pressing and densification of the sheets. However, it was somewhat surprising that, after hot pressing, the ALs, despite having the smallest pores, had a higher air permeance than the FLs and much higher than the WLs (Figure 10). When averaged over all four 60-s hot-pressing conditions, the air permeances were (33 ± 10) mL/min (AL), (10 ± 5) mL/min (FL), and (5 ± 3) mL/min (WL). This trend could not be due to pinholes, because with prolonged pressing of 60 s, the measured air permeance was rather low for the ALs as well, although it remained higher than that of the corresponding FL or WL sheets.

In principle, the ALs could have shown a higher local density variation, as some local regions may have been pressed less than others due to the largest millimeter-scale grammage variation in these sheets. If lower density regions had existed, they should have led to larger pores in the corresponding areas of the AL sample. However, such pores were absent in the narrow pore size distribution of hot-pressed ALs (Figure 8). This suggests that the clearly higher air permeance of the ALs originated from their even microscale structure and low tortuosity^49^ rather than from any macroscopic structural feature.

### Tensile Properties

The tensile properties of fiber network materials are primarily determined by characteristics related to the fibers, the contacts and bonds they make, and the structure of the fiber network. Aspects related to network connectedness include individual bond strength and the degree of overall bonding (relative bonded area). The distribution of fibers (sheet formation) and their orientation, pore structure, and void fraction are some of the key features of the network. Hot pressing applies an external loading on the sheet that directly affects all these aspects, leading to improved tensile strength, as seen in Figure 11.

**Figure 11:**
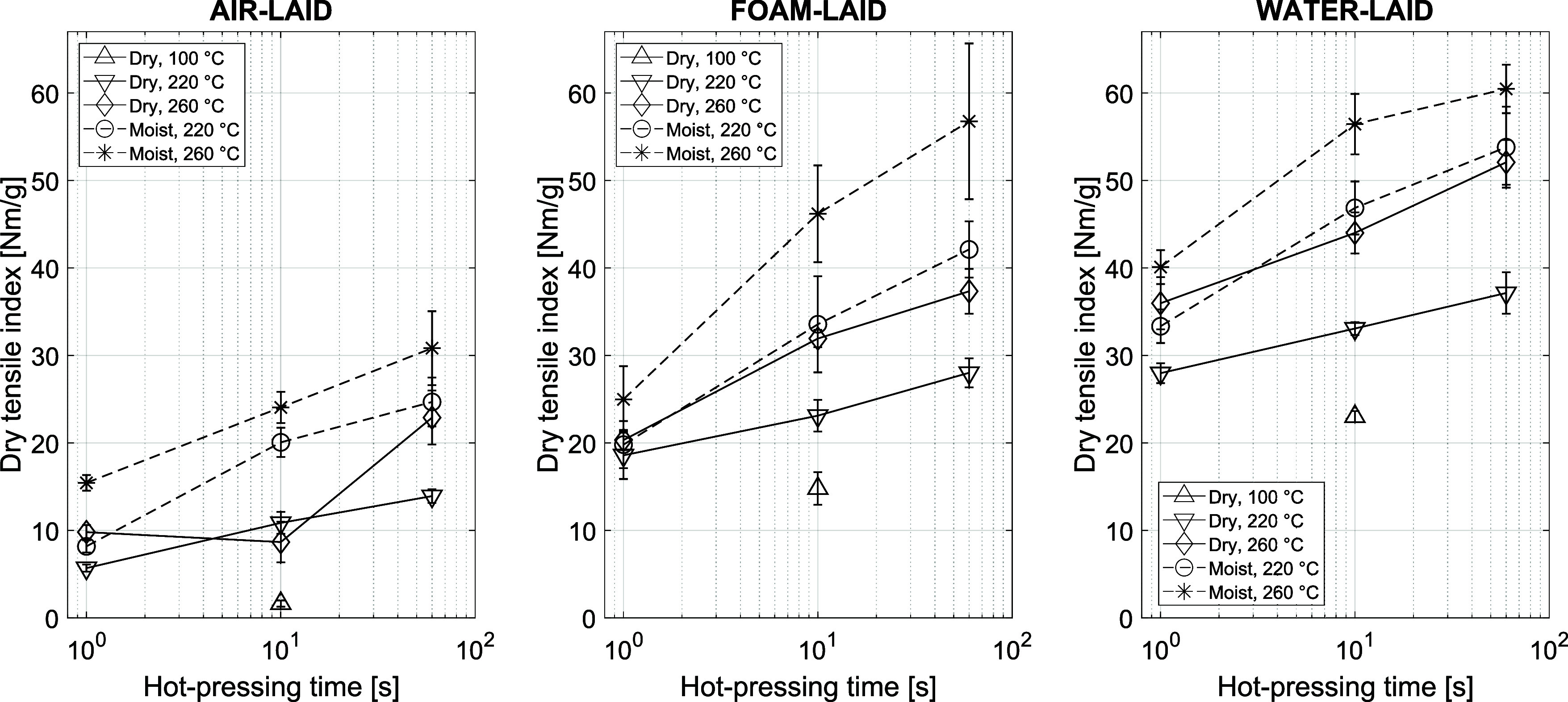
Dry-TI for AL, FL, and WL samples. Unpressed references: FL (14.6 ± 0.8) Nm/g, WL (26.0 ± 1.5) Nm/g.

The dry tensile strength index (Dry-TI) (also called specific strength) continued to increase throughout the hot-pressing time range for all hot-pressing temperatures and both initial moisture contents. The WLs had the best overall strength due to their effective fiber bonding, although fiber distribution had been less uniform than in FLs.^14^ The presence of foam bubbles usually ensures good formation but also reduces the material strength of FLs due to the higher relative volume of large pores.^21^ The ALs had lower strength levels, mainly due to the dry sheet forming process. Strength improvement by lignin interdiffusion^4^ becomes possible when there are enough short-range interfiber contacts in the fiber network. However, the lignin-induced bonding was not enough to compensate for the absence of an efficient bonding regime brought about by the presence of water in the sheet forming.

For sheets made of natural wood fibers, m.c. is a crucial and complex state variable during hot pressing. By nature, water molecules interact with hydrophilic cellulose and hemicelluloses by weakening the hydrogen bonding. As the fibers soften and swell in higher m.c., the pliable fibers compact to densify the sheet (Figure 7). An increasing m.c. reduces the glass transition temperature (*T*_g_) of lignin due to plasticization effects, thus affecting the mechanical properties of the fibers.^50^ Therefore, an increasing m.c. assisted fiber deformation and bonding in the hot-pressing experiment. However, the tensile strength improvement of the ALs remained lower compared to the FLs and WLs.

The sheet density reached its final level during the first 10 s of the compression (see Figure 7), while only half of the total strength improvement was achieved (Figure 11) during this time. This suggests that the fiber network rearrangements occurred first. In the presence of pressure and heat, the lignin and hemicellulose molecules softened, which enabled the collapse of fibers^51^ and other viscoelastic network deformations, leading to sheet densification. At high density, fiber movement is restricted, potentially limiting changes in their orientation and in the structure of interfiber pores. In this case, the number of interfiber bonds is determined solely by density.^52^ The maximum bond number and relative bonded area were thus likely reached after 10 s pressing, potentially also the end point of the pore structural changes.

Hot pressing also improved the TI measured after 1 min of immersion in water (Wet-TI) for all samples, as show in Figure 12. One possible explanation is that hot pressing rendered the interfiber bonds water-resistant from within. This effect could be associated with the partially water-resistant nature of lignin, which increases hydrophobicity at the interphase of bonded fibers. This may have enhanced the bond strength to better tolerate moisture.

**Figure 12:**
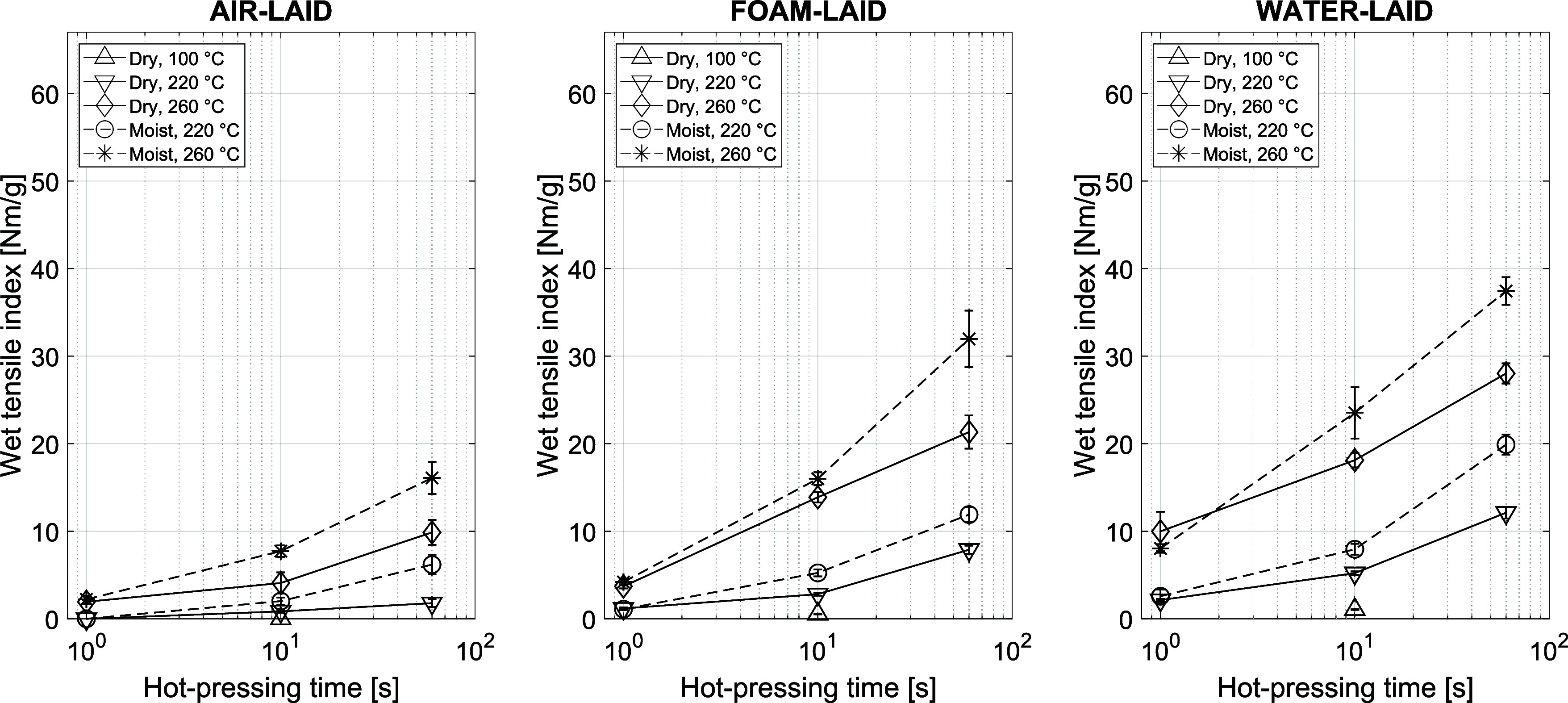
Wet-TI for AL, FL, and WL samples. Unpressed references: FL (0.65 ± 0.03) Nm/g, WL (1.07 ± 0.05) Nm/g.

When the Wet-TI (Supporting Information, Figure S13a) and Dry-TI (Figure 13b) are plotted against density, the data sets are very similar. By offsetting the wet strength values by roughly 20 Nm/g, the two data sets overlap, as shown in Supporting Information, Figure S13b. 20 Nm/g is the average strength level that the FL and WL sheets reached at 100 °C through hydrogen bonding without significant lignin softening, mobilization, and interdiffusion. This strength arises from hydrogen bonds formed during aqueous forming, which are disrupted upon rewetting of the fibers. The new interfiber molecular bonds formed during hot pressing effectively add to the hydrogen bonding. They are equally strong in both dry and wet conditions, indicating the dominant role of lignin in strength improvement in both cases. Similar wet strength increase has not been observed in delignified cellulose materials.^53^ Traditional papermaking does not involve hot-pressing processes. In this context, a high lignin content is usually associated with reduced fiber flexibility and reduced network strength.

**Figure 13:**
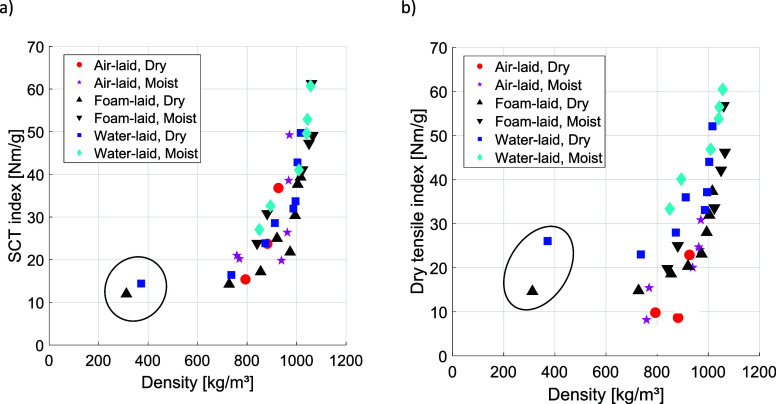
(a) SCT index and (b) Dry-TI against sheet density after hot pressing. Four of the seven AL samples were too weak to sustain in-plane compression, so the corresponding Dry-TI values are removed. The unpressed FL and WL samples (marked with —).

It is also plausible that the exposure to hot pressing reduced the reswelling of the fibers in water. The degree of nearly irreversible association of cellulose, known as hornification, has been found to correlate with the intensity of heat treatment.^54^ Therefore, the fiber–fiber bonds were not subjected to strong expansive stresses. Compared to the FLs and WLs, the ALs reached roughly half the wet strength levels. This can be explained by the originally lower bondedness of those sheets, arising from the microsized gaps in the fiber bond regions of poorly moldable dry fibers.^55^ In addition, the high contact angles measured for the ALs (Table 4) were not reflected in the wet tensile data.

Additional secondary phenomena also may occur in the fiber–fiber contact regions. The yellow-brown coloration (see Figure 4) and the characteristic scent, i.e., the release of volatiles, of the samples indicated such changes. Hemicellulose depolymerization gradually starts at temperatures below 200 °C^56^ and lignin depolymerization at over 200 °C.^57^ However, kinetic studies have reported minimal thermal decomposition for most wood components between 200 and 300 °C,^58^ particularly at exposure times of less than a few minutes. Among these components, xylan is the most thermally labile and begins to decompose already at 200 °C, even during short exposures,^58^ making it a potential contributor following lignin.

### Comparison of In-Plane Compression Strength and Tensile Strength

The suitability of fiber-based materials for packaging applications is often assessed using the in-plane short-span compression test (SCT). This test gives an indication of how well the package can withstand compressive loads without breaking or collapsing. Most properties of the fiber network have a direct influence on the SCT. The SCT index is typically found to be roughly half the TI, as a fiber network tends to better sustain tensile stresses than compressive ones. While the tensile index is mainly governed by material density and interfiber bond strength, shear or buckling deformations of the fiber network can reduce the SCT strength under in-plane compressive stress. For thin samples or long free spans, macroscopic buckling of the test specimen is also possible, which can dominate the measured SCT values. However, this type of failure was not expected in the present study due to the high sheet grammage and the short 0.7 mm free span.^59^

Figure 13 compares the SCT index and the TI as a function of density. The steep rise in both parameters beyond a density of 800 kg/m^3^ is related to the application of high temperatures (220 °C or above) during pressing. However, for the dry AL sample at 220 °C, pressing also had to be longer than 10 s before significant tensile or compressive strength could be achieved. In other words, mere sample densification and formation of new interfiber contacts did not increase strength; a strength increase required bonding of the fibers through polymer (lignin) interdiffusion. This behavior differed from that of the FL and WL samples, which were already well bonded prior to hot pressing. Interestingly, even for these samples, the fiber–fiber contacts formed by densification did not add strength until they became bonded via the same interdiffusion mechanism. This resulted in highly nonlinear SCT and tensile index behaviors.

The strength results were comparable for both aqueous forming methods, with a slightly higher TI obtained for the WL samples under the given pressing conditions. Both TI and SCT were higher if the sheets were moist before the hot-pressing operation. This was partly explained by the more effective densification of the samples achieved with softer fibers. However, the fact that different strength values were obtained at equal density indicates that the quality of the interfiber bonds also played an important role.

Notably, while the TI values varied greatly with the pressing conditions, the SCT was less sensitive to both pressing conditions and forming method. This is to be expected, as compressive loads (SCT) tend to average out structural deformations across the sheet, whereas tensile loads (TI) concentrate stresses near the weakest points.

Interestingly, when hot-pressed in the dry state, the AL samples reached an SCT level comparable to that of the other forming methods at the same density, despite exhibiting clearly lower TI. This suggests that, beyond a certain threshold strength of the interfiber bonds, the SCT index is dominated by fiber stiffness and shear and buckling deformations rather than bond opening. Moreover, network-level deformations under in-plane compression appear similar for all forming methods, likely due to the high material density and the very large relative bonded area after hot pressing. The densified structure is also the probable reason why the SCT values are comparable to those of TI, which is not normally the case with lower-density sheets. In previous studies, creep compliance in tension and compression coincided at high, but not low, sheet densities.^46,60^

Finally, from an application standpoint, the proposed hot-pressing method shows potential for scaling to industrial processes. This would involve transitioning from discontinuous to continuous pressing. These operation modes differ fundamentally in aspects such as heat transfer mechanisms, pressing time, and process kinetics. However, previous studies on continuous hot pressing at both laboratory and industrial scales have shown similar systematic trends with respect to pressing time and temperature as those obtained with discontinuous hot pressing.^53,61^ At the same time, discontinuous hot pressing remains relevant for forming various fiber-based blanks into three-dimensional structures, such as trays.

## Conclusion

This study explored the impact of three web-forming methods—air-laid, foam-laid, and water-laid—on sheet properties under intense hot-pressing conditions (5 MPa, 100–260 °C, 1–60 s). These methods differ in the amount of water available for native bonding of wood fibers, as well as in the continuity and viscosity of the forming medium. The study also showed how the response of lignin-rich fibers to high temperature, pressure, and initial m.c. affected sheet properties after hot pressing.

The characteristics of the forming medium—air, water, and wet foam—were consistently retained through the drastic hot-pressing process and influenced the final structure and material properties. Despite the very high final densities, ranging from 800 kg/m^3^ to 1000 kg/m^3^, traces of the original differences in the porous structures could still be seen in the number of large pores in the pressed sheets and in the overall homogeneity of the material. The number of larger pores was the lowest for the air-laid sheets, followed by the water-laid and foam-laid sheets. Even so, the air-laid sheets had the highest air permeance, which could be associated with their well-dispersed microscale distribution of fibers leading to low tortuosity. In that respect, thin hot-pressed air-laid materials may be suitable for filtration applications, either as such or as a permeable substrate for an electrospun polymer layer.^62^ In general, filter media made of biodegradable and hydrophilic wood fibers may be suitable for air or dust filtration. Furthermore, the air-laid sheets exhibited higher contact angles compared to both the foam-laid and water-laid sheets, despite having lower density and strength values, as well as lower SCT values.

Hot pressing improved tensile strength due to considerable sheet densification and enhanced fiber bonding. The strength improvement continued beyond the first 10 s, during which the main structural changes in the fiber network were considered to have been completed. Thus, the strength improvement was due to dynamic polymer-level changes in the sheets, especially in the interfiber contact areas. Wet tensile strength was enhanced by hot pressing due to water-resistant fiber–fiber bonds reinforced with lignin at high temperature. The air-laid-samples showed lower dry and wet tensile strength due to less effective network formation and lack of original bondedness prior to hot pressing.

## Supplementary Material



## Notes

Additional method descriptions and result graphs are provided in the Supporting Information file. Cross-references to its figures are labeled with an “S” (PDF)

## References

[1] Joelsson T., Pettersson G., Norgren S., Svedberg A., Höglund H., Engstrand P. (2020). High strength paper from high yield pulps by means
of hot-pressing. Nord Pulp Pap Res. J..

[2] Koljonen K., Österberg M., Johansson L. S., Stenius P. (2003). Surface chemistry and
morphology of different mechanical pulps determined by ESCA and AFM. Colloid Surface A.

[3] Börås L., Gatenholm P. (1999). Surface composition
and morphology of CTMP fibers. Holzforschung.

[4] Elf P. (2025). Role of lignin in hot-pressing of paper: Insights from
molecular
simulations and experiments. Biomacromolecules.

[5] Mattsson A., Joelsson T., Miettinen A., Ketoja J. A., Pettersson G., Engstrand P. (2021). Lignin inter-diffusion underlying improved mechanical
performance of hot-pressed paper webs. Polymers.

[6] Michielsen S., Pourdeyhimi B., Desai P. (2006). Review of thermally
point-bonded
nonwovens: Materials, processes, and properties. J. Appl. Polym. Sci..

[7] Hou S. Y., Wang J. Y., Yin F. Y., Qi C. S., Mu J. (2022). Moisture sorption
isotherms and hysteresis of cellulose, hemicelluloses and lignin isolated
from birch wood and their effects on wood hygroscopicity. Wood Sci. Technol..

[8] Gasparovic L., Korenová Z., Jelemensky L. (2010). Kinetic study of wood chips decomposition
by TGA. Chem. Pap..

[9] Börcsök Z., Pásztory Z. (2021). The role of
lignin in wood working processes using
elevated temperatures: An abbreviated literature survey. Eur. J. Wood Prod..

[10] Kojiro K., Furuta Y., Ishimaru Y. (2008). Effects of
heating from 100°C
to 200°C on dynamic viscoelastic properties of dry wood. J. Soc. Mat. Sci..

[11] Felhofer M., Bock P., Singh A., Prats-Mateu B., Zirbs R., Gierlinger N. (2020). Wood deformation
leads to rearrangement
of molecules at the nanoscale. Nano Lett..

[12] Oliaei E., Berthold F., Berglund L. A., Lindström T. (2021). Eco-friendly
high-strength composites based on hot-pressed lignocellulose microfibrils
or fibers. Acs Sustain Chem. Eng..

[13] Smith M. K., Punton V. W., Rixson A. G. (1974). Structure
and properties of paper
formed by a foaming process. Tappi.

[14] Hjelt T., Ketoja J. A., Kiiskinen H., Koponen A. I., Pääkkönen E. (2022). Foam forming
of fiber products: A review. J. Disper Sci.
Technol..

[15] Hirn U., Schennach R. (2015). Comprehensive analysis of individual
pulp fiber bonds
quantifies the mechanisms of fiber bonding in paper. Sci. Rep..

[16] Brydon, A. G. ; Pourmohammadi, A. ; Russell, S. J. Chapter 4: Drylaid web formation. In Handbook of Nonwovens; Elsevier, 2022; pp. 89–181.

[17] Rawal A., Rao P. V. K., Russell S., Jeganathan A. (2010). Effect of
fiber orientation on pore size characteristics of nonwoven structures. J. Appl. Polym. Sci..

[18] Paunonen S., Keränen J. T., Kamppuri T. (2024). Air-laid and foam-laid nonwoven composites:
The effect of carrier medium on mechanical properties. J. Appl. Polym. Sci..

[19] Kononov, A. ; Drobosyuk, V. ; Paulapuro, H. , “Application of the air dynamic forming method for coarse mechanical pulp,” In 58th Appita Annual Conference and Exhibition Incorporating the Pan Pacific Conference, Incorporating the Pan Pacific Conference: Canberra, Australia 2004, pp. 97–102.

[20] Notley S. M., Norgren M. (2010). Surface energy and wettability of spin-coated thin
films of lignin isolated from wood. Langmuir.

[21] Al-Qararah A. M., Hjelt T., Kinnunen K., Beletski N., Ketoja J. A. (2012). Exceptional
pore size distribution in foam-formed fibre networks. Nord Pulp Pap Res. J..

[22] Al-Qararah A. M. (2015). A unique microstructure
of the fiber networks deposited from foam-fiber
suspensions. Colloid Surface A.

[23] ISO ISO 16065–1 Pulps — Determination of fibre length by automated optical analysis — Part 1: Polarized light method; ISO, 2001.

[24] Lappalainen L., Lehmonen J. (2012). Determinations of bubble
size distribution of foam-fibre
mixture using circular hough transform. Nord
Pulp Pap Res. J..

[25] ISO ISO 5269–1 Pulps-Preparation of laboratory sheets for physical testing, conventional sheet- former method; ISO, 2005.

[26] Negro C., Pettersson G., Mattsson A., Nyström S., Sanchez-Salvador J. L., Blanco A., Engstrand P. (2023). Synergies
between fibrillated nanocellulose and hot-pressing of papers obtained
from high-yield pulp. Nanomaterials.

[27] Fellers, C. ; Norman, B. ; Westerlund, Ö. , Pappersteknik. Kungliga Tekniska högskolan Institutionen för pappers- och massateknik: Stockholm, Sweden, 1996, pp. 432.

[28] Sakairi, K. ; Ishii, M. , Molded pulp article and method for producing molded pulp article. US 12,195,924 B2, 2024.

[29] Freville E., Sergienko J. P., Mujica R., Rey C., Bras J. (2024). Novel technologies
for producing tridimensional cellulosic materials for packaging: A
review. Carbohyd Polym..

[30] ISO 187 Paper, board and pulps — Standard atmosphere for conditioning and testing and procedure for monitoring the atmosphere and conditioning of samples. ISO, 1990.

[31] ISO 534:2011 Paper and board - Determination of thickness, density and specific volume, ISO 2011.

[32] ISO 536:2019 Paper and board - Determination of grammage, ISO, 2019.

[33] ISO 5636–3:2013; Paper and Board—Determination of air permeance (medium range)—Part 3: Bendtsen method; ISO: Geneva, Switzerland, 2013.

[34] ISO ISO 1924–3:2005 Paper and board — Determination of tensile properties, Part 3: Constant rate of elongation method (100 mm/min); 2005.

[35] ISO 9895:2008 Paper and board - Compressive strength – Short-span test; ISO, 2008.

[36] Ketola A.
E. (2022). Changing
the structural and mechanical anisotropy of foam-formed
cellulose materials by affecting bubble-fiber interaction with surfactant. Acs Appl. Polym. Mater..

[37] Hubbe M. A., Pizzi A., Zhang H. Y., Halis R. (2017). Critical links governing
performance of self-binding and natural binders for hot-pressed reconstituted
lignocellulosic board without added formaldehyde: A Review. Bioresources.

[38] Berg, J. C. Chapter V - The role of surfactants Textile science and technology 13 - Absorbent Technology. Chatterjee, P. K. ; Gupta, B. S. Eds.; Elsevier, 2002.

[39] Law K. Y. (2014). Definitions
for hydrophilicity, hydrophobicity, and superhydrophobicity: Getting
the basics right. J. Phys. Chem. Lett..

[40] Salmén L. (1984). Viscoelastic
properties of in situ lignin under water-saturated conditions. J. Mater. Sci..

[41] Paajanen A., Zitting A., Rautkari L., Ketoja J. A., Penttila P. A. (2022). Nanoscale
mechanism of moisture-induced swelling in wood microfibril bundles. Nano Lett..

[42] Stenström S. (2020). Drying of
paper: A review 2000–2018. Dry Technol..

[43] Pounder, J. R. ; Ahrens, F. W. ; A Mathematical model of high intensity paper drying, Doctoral dissertation; The institute of paper chemistry, Lawrence university, Appleton, WI, 1986.

[44] Niskanen K., Simula S. (1999). Thermal diffusivity
of paper. Nord Pulp Pap Res. J..

[45] Krook R., Stenstrom S. (1998). Temperature
gradients and heat flux measurements in
hot pressing of paper. Exp Heat Transfer.

[46] Coffin, D. W. , “The creep response of paper. In Advances in Paper Science and Technology: Transactions of the 13th Fundamental Research Symposium; 13th Fundamental Research Symposium, 2005, pp. 651–747.

[47] Brezinski J. P. (1956). The creep
properties of paper. Tappi J..

[48] Lehmonen J., Retulainen E., Paltakari J., Kinnunen-Raudaskoski K., Koponen A. (2020). Dewatering
of foam-laid and water-laid structures and
the formed web properties. Cellulose.

[49] Koponen A., Ekman A., Mattila K., Al-Qararah A. M., Timonen J. (2017). The Effect of void structure on the permeability of
fibrous networks. Transport Porous Med..

[50] Bouajila J., Dole P., Joly C., Limare A. (2006). Some laws of a lignin
plasticization. J. Appl. Polym. Sci..

[51] Pasquier E., Skunde R., Ruwoldt J. (2023). Influence
of temperature and pressure
during thermoforming of softwood pulp. J. Bioresour
Bioprod..

[52] Komori T., Makishima K. (1977). Numbers of fiber-to-fiber contacts
in general fiber
assemblies. Text. Res. J..

[53] Joelsson, T. , The influence of pulp type and hot-pressing conditions on paper strength development, Doctoral, Department of Chemical Engineering; MoRe Research Örnsköldsvik AB., Mid Sweden University, 2021.

[54] Sellman F.
A., Benselfelt T., Larsson P. T., Wagberg L. (2023). Hornification of cellulose-rich
materials – A kinetically trapped state. Carbohydr. Polym..

[55] Sormunen T., Ketola A., Miettinen A., Parkkonen J., Retulainen E. (2019). X-Ray nanotomography of individual
pulp fibre bonds
reveals the effect of wall thickness on contact area. Sci. Rep..

[56] Varol E. A., Mutlu Ü. (2023). TGA-FTIR analysis
of biomass samples based on the thermal
decomposition behavior of hemicellulose, cellulose, and lignin. Energies.

[57] Tan H., Wang S.-R., Luo Z.-Y., Cen K.-F. (2006). Pyrolysis behavior
of cellulose, xylan and lignin. J. Fuel Chem.
Technol..

[58] Chen W. H., Kuo P. C. (2011). Isothermal torrefaction kinetics of hemicellulose,
cellulose, lignin and xylan using thermogravimetric analysis. Energy.

[59] Pelczynski P., Szewczyk W., Bienkowska M., Kolakowski Z. (2023). A New technique
for determining the shape of a paper sample in in-plane compression
test using image sequence analysis. Appl. Sci..

[60] Vorakunpinij, A. , The effect of paper structure on the deviation between tensile and compressive creep responses, Ph.D. Dissertation; Institute of Paper Science and Technology, Georgia Institute of Technology, Atlanta, GA, 2003.

[61] Rafi A. A. (2025). Continuous Fabrication of Strong, Scalable, High-Yield,
and Sustainable
Materials from Aspen. Acs Sustain Chem. Eng..

[62] Ketoja J. A. (2024). Design of biodegradable cellulose filtration material with high efficiency
and breathability. Carbohyd Polym..

