# Supplementary material for: Property Variations of Binder-Free Lignin-Rich Fiber Networks Driven by Forming Processes and Hot Pressing
Source: ACS Omega. 2026 Jan 25;11(5):7129–41. doi: 10.1021/acsomega.5c06266 (PMC12903141; doi:10.1021/acsomega.5c06266)
Supplement: Supplementary file 1 [file ao5c06266_si_001.pdf]

Supporting Information for:

Property Variations of Binder-Free Lignin-Rich Fiber Networks Driven by Forming Processes and Hot Pressing

Sara Paunonen*, Amanda Mattsson, Gunilla Pettersson, Jukka A. Ketoja

Chemical composition of CTMP fibers

To determine the carbohydrate and lignin composition, fiber samples were hydrolyzed with sulfuric acid, and the resulting monosaccharides were determined by high-performance anion-exchange chromatography (HPAEC) with pulse amperometric detection (Dionex ICS 5000, equipped with a CarboPac PA20 column).

The polysaccharide content in the samples was calculated from the corresponding monosaccharides using an anhydrous correction of 0.88 for pentoses and 0.9 for hexoses [1]. The Klason lignin content, i.e., the insoluble residue from hydrolysis, was determined gravimetrically. The acid-soluble lignin in the hydrolysate was quantified by ultraviolet spectroscopy at 215 nm and 280 nm, as described by [2]. The cellulose and hemicellulose fractions were calculated based on [3]. Two repetitions were carried out.

Sheet grammage and thickness after hot pressing

Figure S1 shows the measured grammages (target 450 g/m^2). The largest variation was observed for the ALs that were cut directly from pilot rolls, whereas the FL and WL laboratory sheets showed only minor deviations. These small variations were not expected to significantly affect the results.

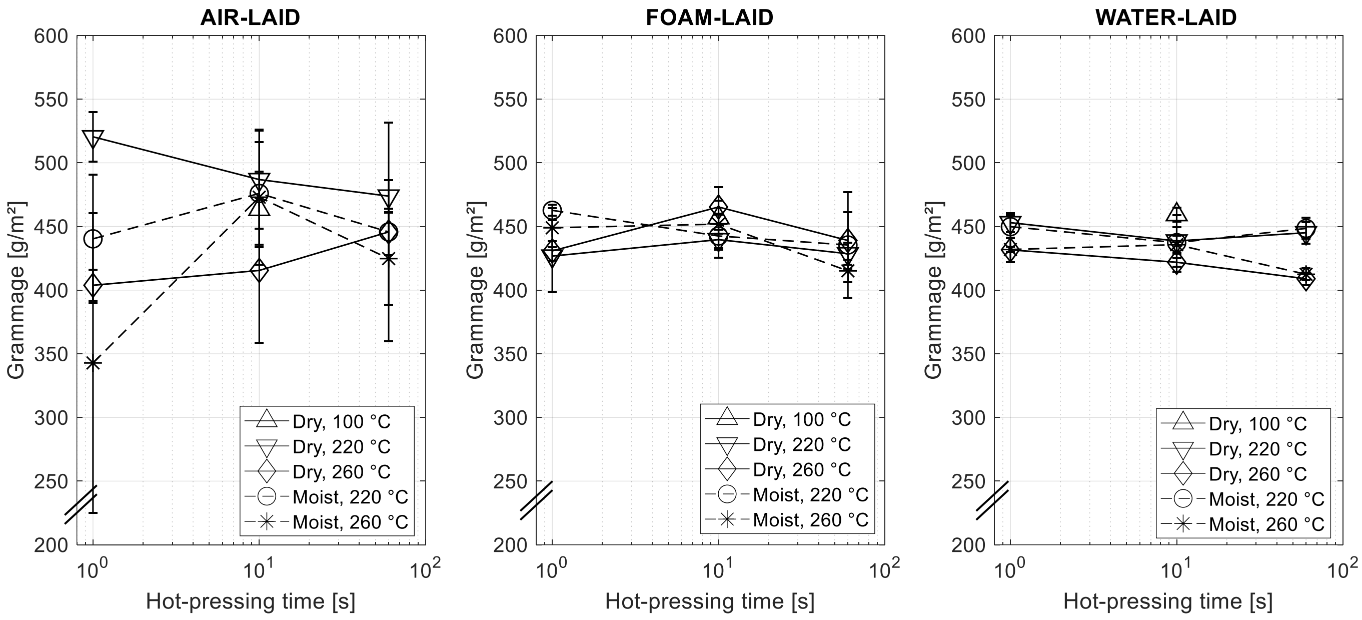

Figure S1. Sheet grammages in various hot-pressing tests. Average ± SD. Unpressed references: FL (460 ± 8) g/m², WL (450 ± 9) g/m².

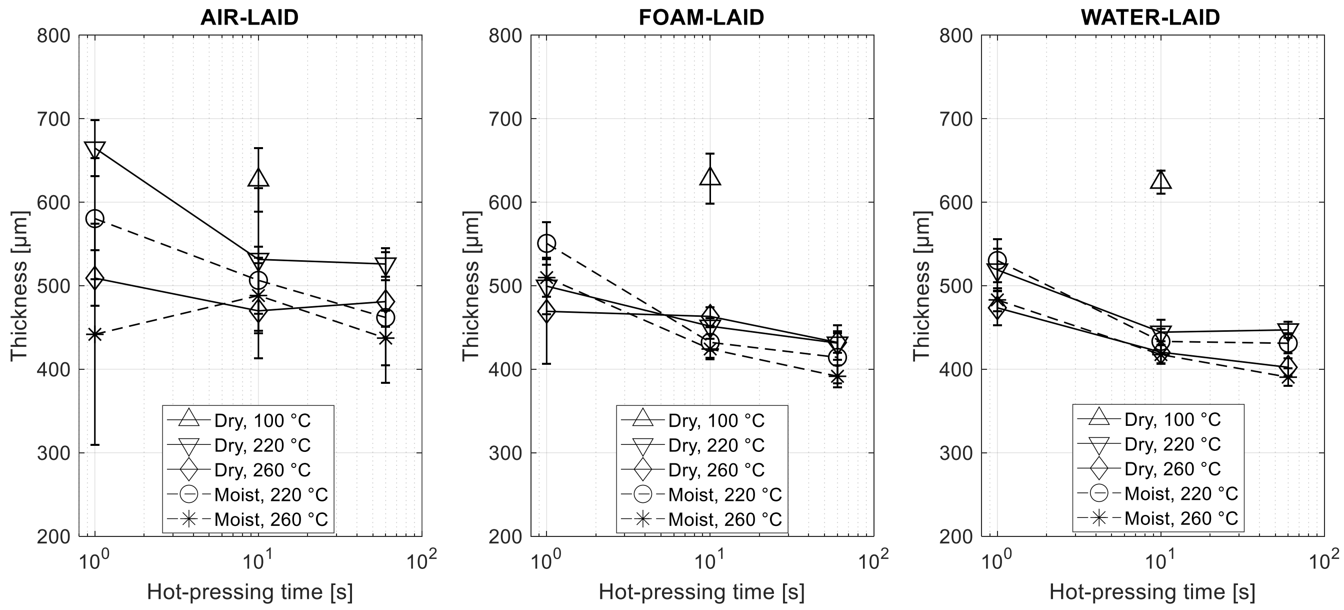

Figure S2. Sheet thicknesses in various hot-pressing tests. Average ± SD. Unpressed references: FL (1460 ± 50) µm, WL (1210 ± 40) µm.

Microporous structural analysis with X-ray microtomography

Porosity

Porosity (void fraction) strongly affects the material's mechanical, physical, and functional properties. According to Figure S3, no significant differences in porosity were observed between the sheet types under the same compression conditions. Increasing temperature made the wood fibers more pliable, leading to reduced porosity. Higher moisture content further reduced porosity.

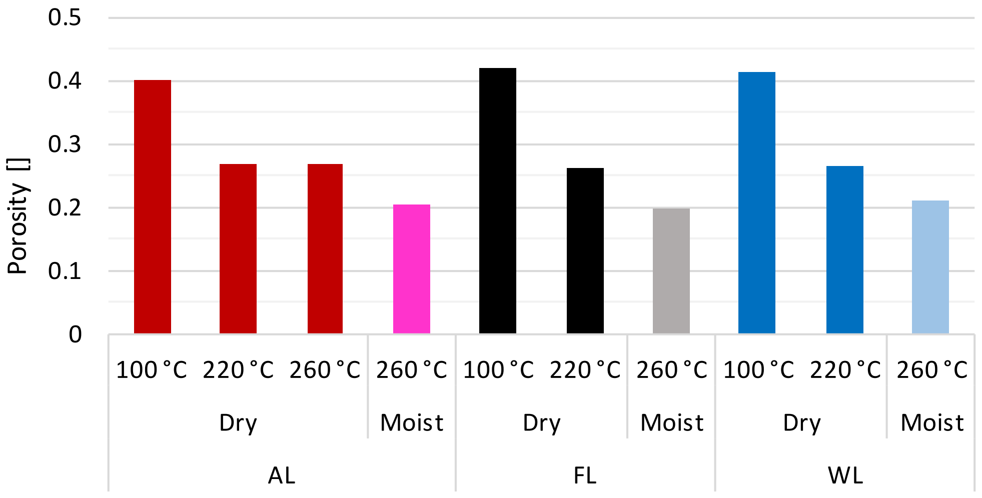

Figure S3. Average sheet porosity determined from two CT scans per material type and hot-pressing condition.

Interestingly, the measured internal porosity was not affected by the forming method, even though the AL sheets had a systematically slightly lower density than the FLs and WLs (Figure S3). This indicates that the measured density variation originated from the grammage variation in the ALs, rather than from differences in the void fractions in sheets made by different forming methods.

Pore size analysis

X-ray microtomography (Figure S4) shows that most pores in the hot-pressed sheets were relatively small, with characteristic pore diameters of 10 µm to 20 µm, corresponding to typical CTMP fiber width. For moist fibers pressed at 260 °C, lumens and inter-fiber pores collapsed, resulting in a sharp peak around 10 µm (Figure S4), especially for the ALs. This suggests that the ALs had a more homogeneous fiber network structure than the WLs and FLs. Figure S5 shows that severe pore collapse cannot be achieved through high pressing temperature alone when fibers are dry before pressing.

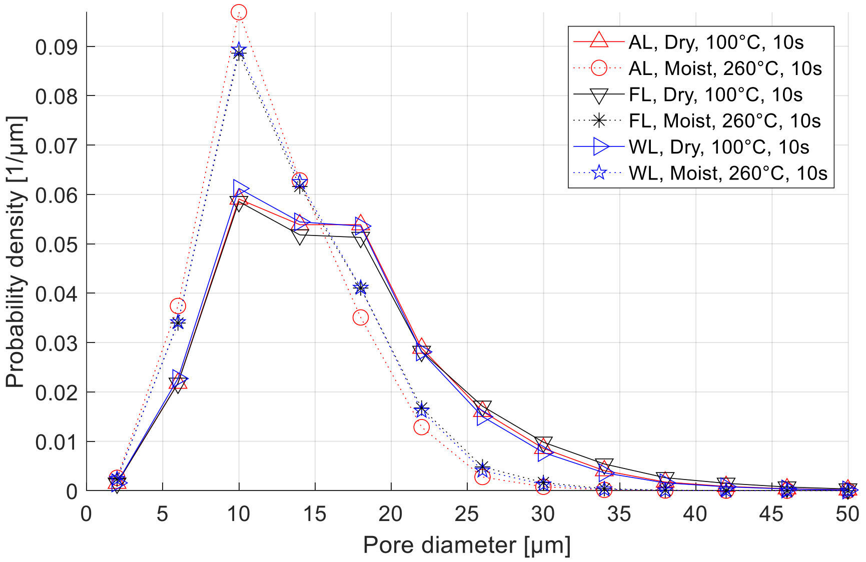

Figure S4. Probability density of pore diameter in thickness direction of AL, FL, and WL under mild (Dry, 100 °C) and severe (Moist, 260 °C) hot pressing conditions. Data averaged from two scans per condition.

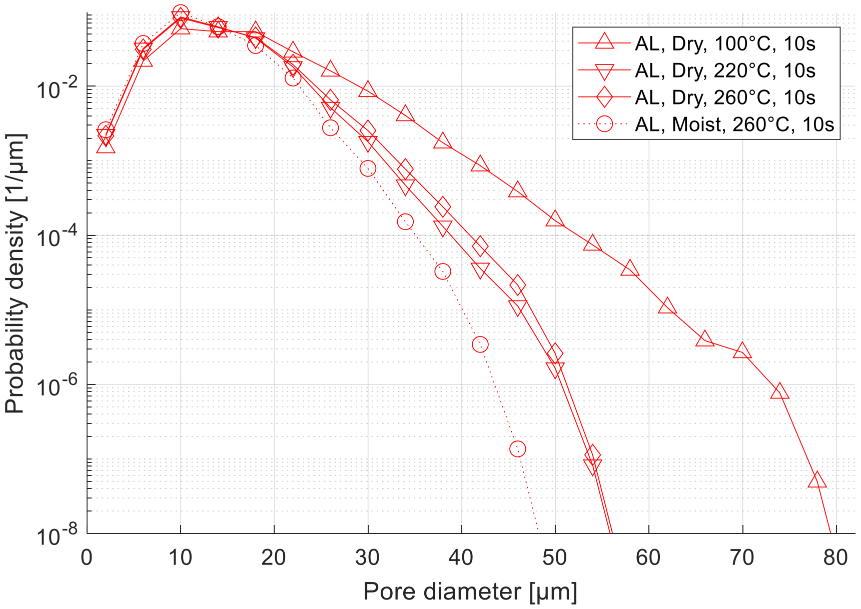

Figure S5. PDF of pore diameters of the ALs. Data averaged from two scans per condition.

The behavior of the largest pores in the FL sheets is also of interest (Figure S6). The mean bubble size of the aqueous foam introduced a small fraction of pores around 70 µm in diameter under mild pressing conditions. Although the overall distribution narrowed with increasing temperature, these large pores remained

distinguishable, giving the distribution a different shape than that of the ALs (Figure S5). However, moistening the fibers before high-temperature pressing reduced this effect.

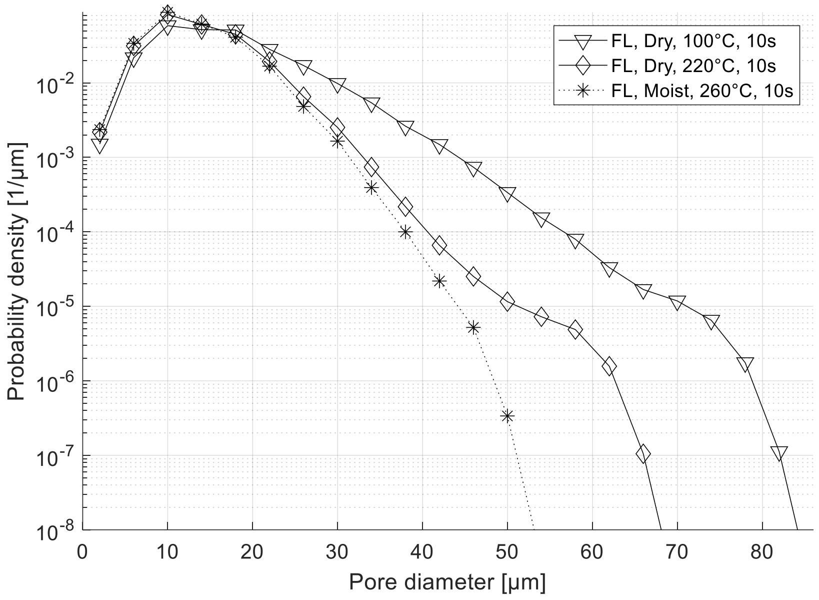

Figure S6. PD of pore diameter of foam-laid sheets. Data averaged from two CT scans per condition.

Pore size profile in the thickness direction

For the dry sheets pressed at 100 °C, the volume-weighted average pore diameter profiles were relatively uniform (Figure S7). Slightly larger average pores were seen on the top side, especially in the AL samples. In contrast, higher pressing temperature resulted in clearly smaller pores in the sheet center for the ALs. This may have been caused by viscoelastic time-dependent structural changes that vary across the sheet thickness. Faster drying at the surface in direct contact with the hot plate may have delayed heating and pore compression on the opposite side.

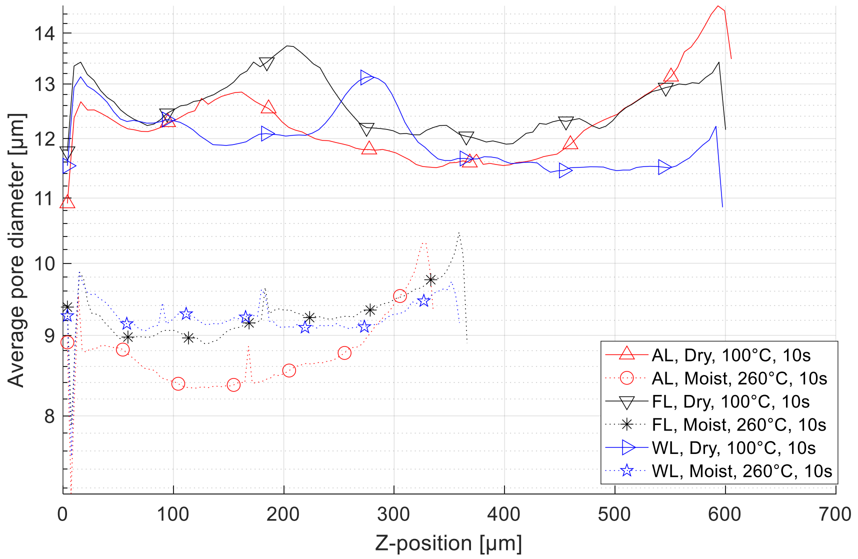

Figure S7. Pore size profile in sheet thickness direction. Each curve is averaged from two CT scans per condition.

Optical formation

Floc size calculation

Grayscale images were thresholded using the median value of the grayscale distribution to distinguish lighter and darker regions. The binarized images were then analyzed using a run-length encoding (RLE) technique. This method compressed sequences of identical pixels, allowing for an efficient calculation of flock size.

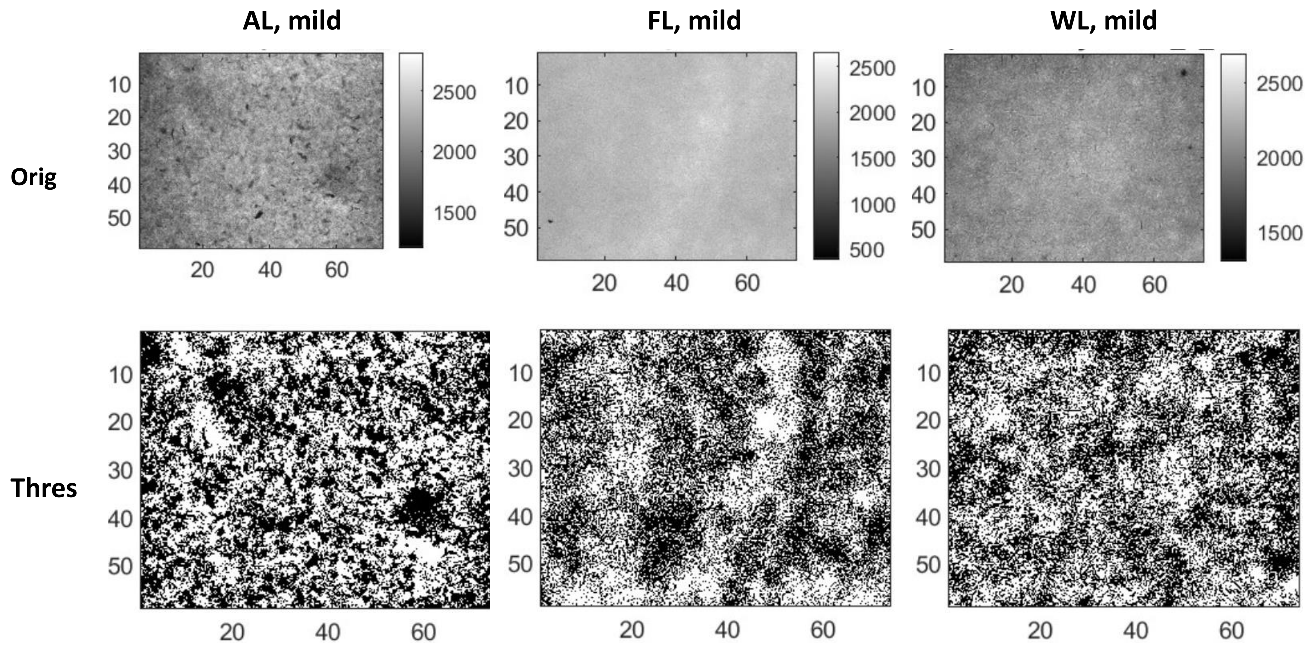

Figure S8. Original (Orig), and median-thresholded images (Thres) for analyzing optical formation.

Table S1 shows additional parameters that highlight distinct characteristics, especially the structural unevenness of the ALs compared to the FLs and WLs.

Table S1. Optical formation parameters.

	Skewness	Kurtosis	Spec	COV	COV/35mm
AL, mild	2083	197	3.08	0.095	0.07
FL, mild	2054	90	1.58	0.044	0.03
WL, mild	2072	107	1.88	0.051	0.04

Surface structural features with SEM imaging

Figure S9 shows that AL fibers hot-pressed at 100 °C appear more rounded (thick) than FL and WL fibers. This difference disappears at 260 °C. At 220 °C (Figure S10), the AL fibers still show a similar rounded shape and a more open surface compared to the FL and WL samples.

**AIR-LAID**	**FOAM-LAID**	**WATER-LAID**
a) Dry, 100 °C, 10 s	b) Dry, 100 °C, 10 s	c) Dry, 100 °C, 10 s

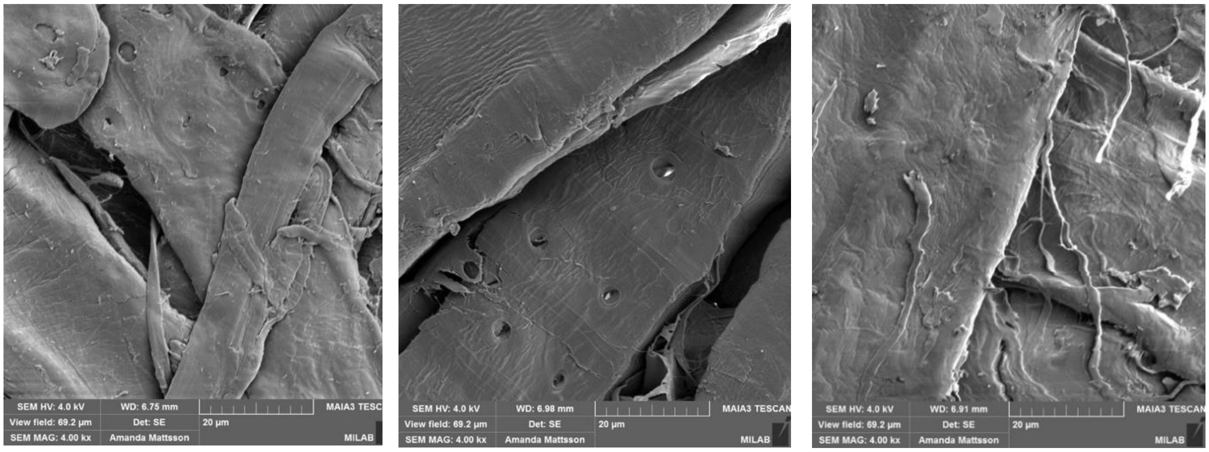

d) Moist, 260 °C, 10 s	e) Moist, 260 °C, 10 s	f) Moist, 260 °C, 10 s

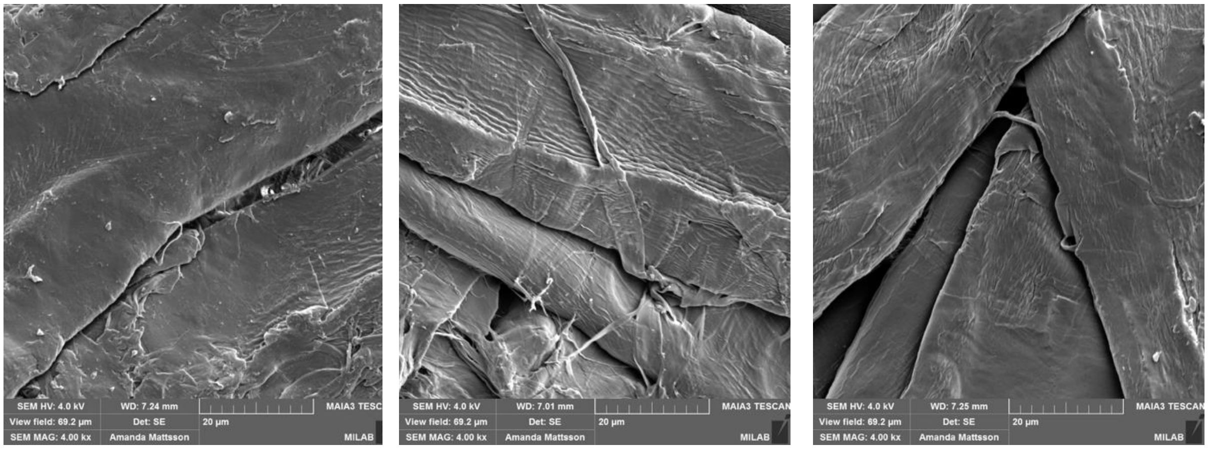

Figure S9. SEM images of sheet surfaces under mild (a, b, c) and intense (d, e, f) hot pressing conditions. Magnification 2000x.

**AIR-LAID**	**FOAM-LAID**	**WATER-LAID**
a) Dry, 220 °C, 10 s	b) Dry, 220 °C, 10 s	c) Dry, 220 °C, 10 s

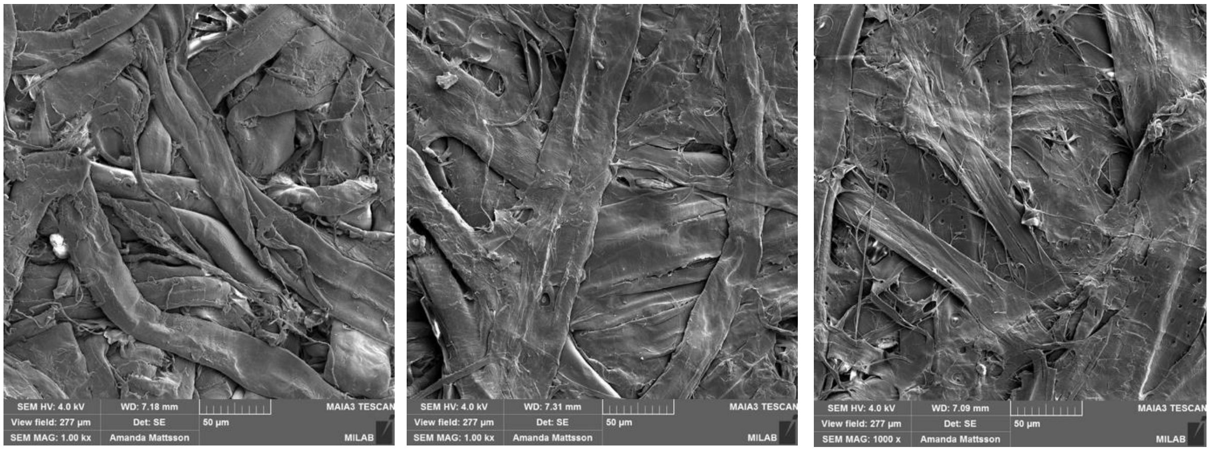

d) Dry, 220 °C, 10 s	e) Dry, 220 °C, 10 s	f) Dry, 220 °C, 10 s

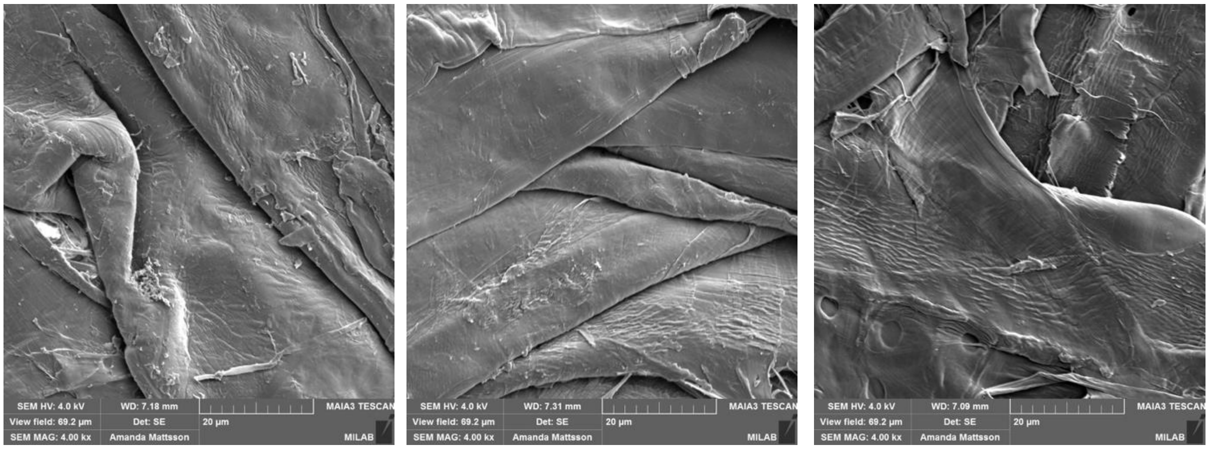

Figure S10. SEM images of sheet surfaces under mild conditions. Magnifications of 500x (a, b, c) and 2000x (d, e, f).

Mechanical properties

Dry tensile test

According to Figure S11, dry strain-at-break clearly depended on the forming method but was hardly affected by the hot-pressing conditions. This demonstrates that the fiber network, established during the forming operation, influenced the final material properties even after major material densification. In contrast, dry tensile stiffness (Figure S12) was greatly affected by material densification.

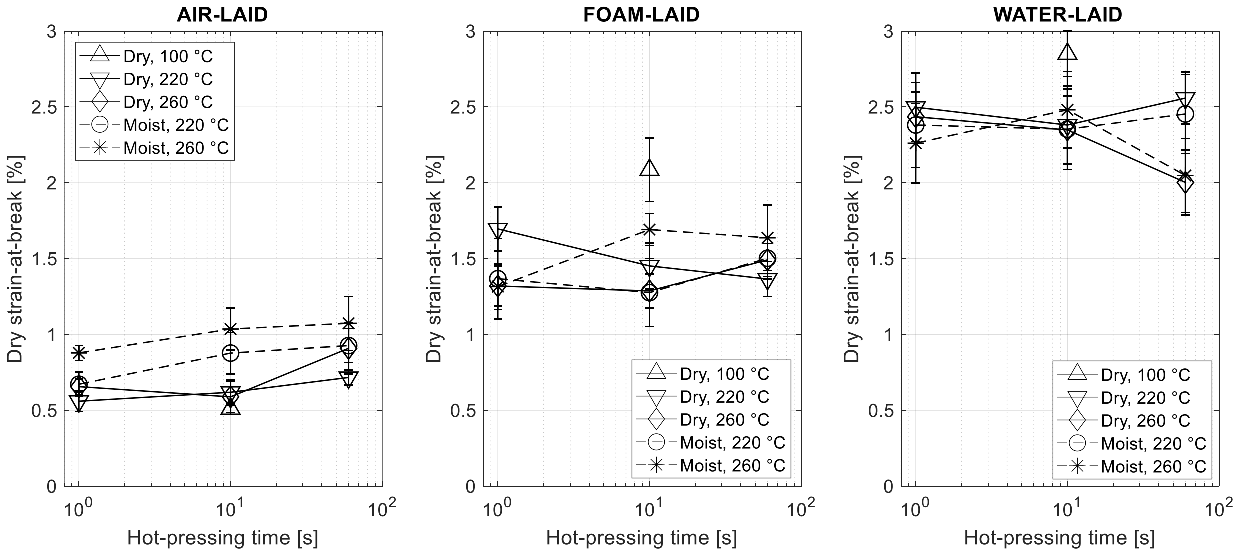

Figure S11. Strain-at-break from the dry tensile test. Unpressed references: FL (1.2 ±0.06) %, WL (2.3 ± 0.2) %.

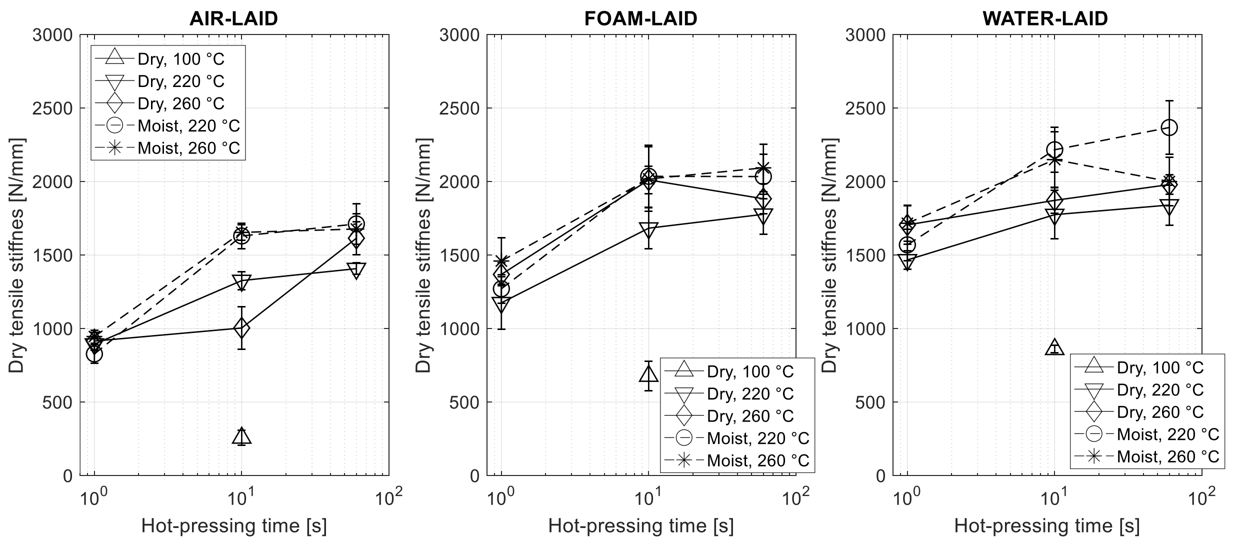

Figure S12. Tensile stiffness from the dry tensile test. Unpressed references: FL (910 ± 70) N/mm, WL (1140 ± 80) N/mm.

Wet tensile strength index

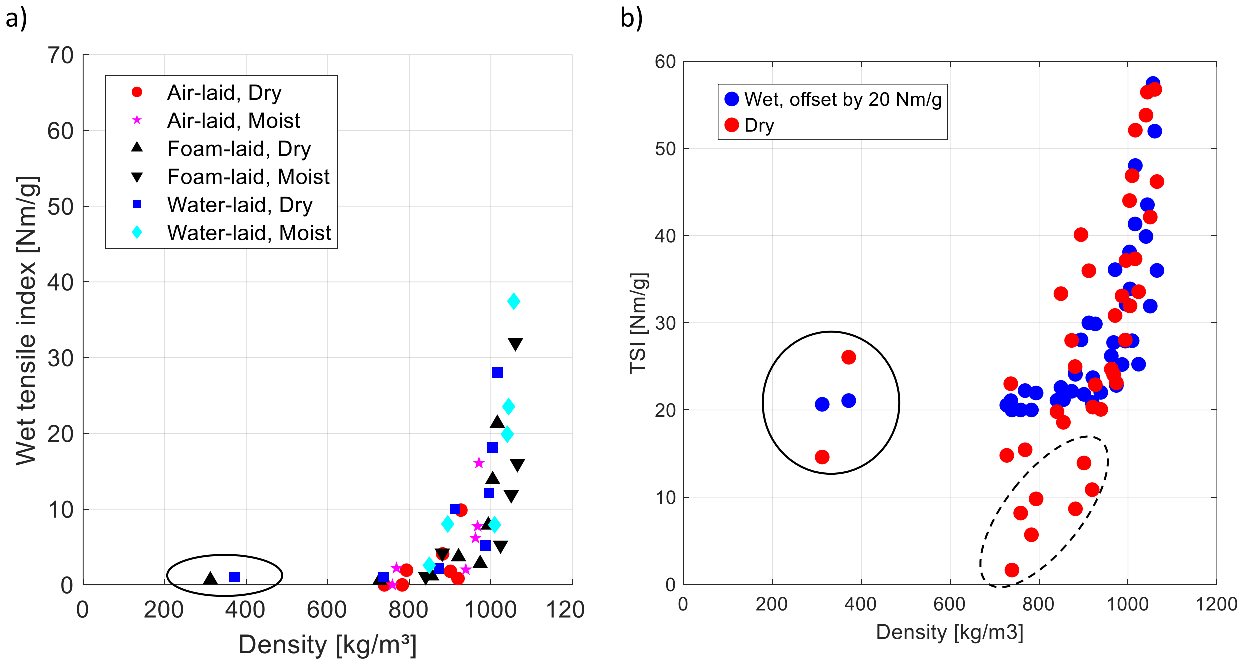

Figure S13. (a) Wet-TSI against final sheet density. (b) The Wet-TSI data was offset by 20 Nm/g and plotted together with the Dry-TSI data. The unpressed FL and WL samples (—). AL samples (- - -).

When plotted against sheet density, both Wet-TSI (Figure S13a) and Dry-TSI (Figure 12b, the journal article) show a steep rise above 800 kg/m^3. At this density, the fiber-fiber contacts were intimate and lignin softening at high temperatures likely caused interdiffusion [4] across the interfaces. The main difference is the onset strength of this increase. Below 800 kg/m^3, Wet-TSI was practically negligible, whereas Dry-TSI of the unpressed WL and FL samples remained at approximately 20 Nm/g, due to hydrogen bonding formed during aqueous sheet formation. By shifting the wet strength values by 20 Nm/g, the wet and dry strength data overlap (Figure

S13b). This comparison does not apply to wet strain-at-break (Figure S14) or wet tensile stiffness (Figure S15), as these depend not only on bonding, but also on the elastic modulus and inelastic deformations of fibers.

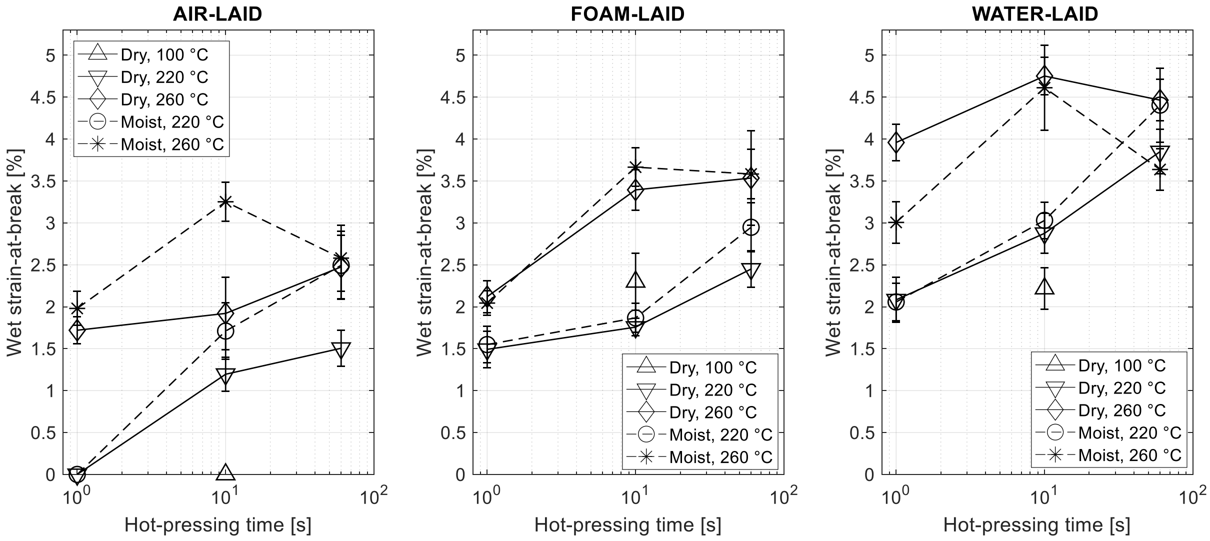

Figure S14. Strain-at-break from the wet tensile test. Unpressed references: FL (1.7 ± 0.2) Nm/g, WL (2.1 ± 0.3) Nm/g.

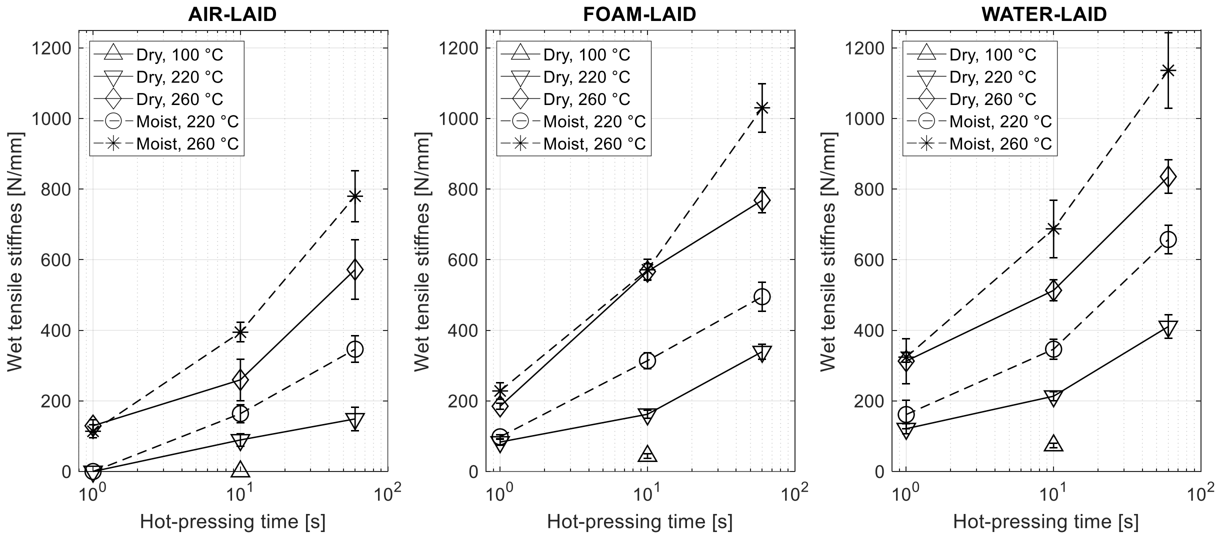

Figure S15. Tensile stiffness from the wet tensile test. Unpressed references: FL (59 ± 5) Nm/g, WL (79 ± 7) Nm/g.

Compression properties

Figure S16 shows the SCT index against hot pressing time.

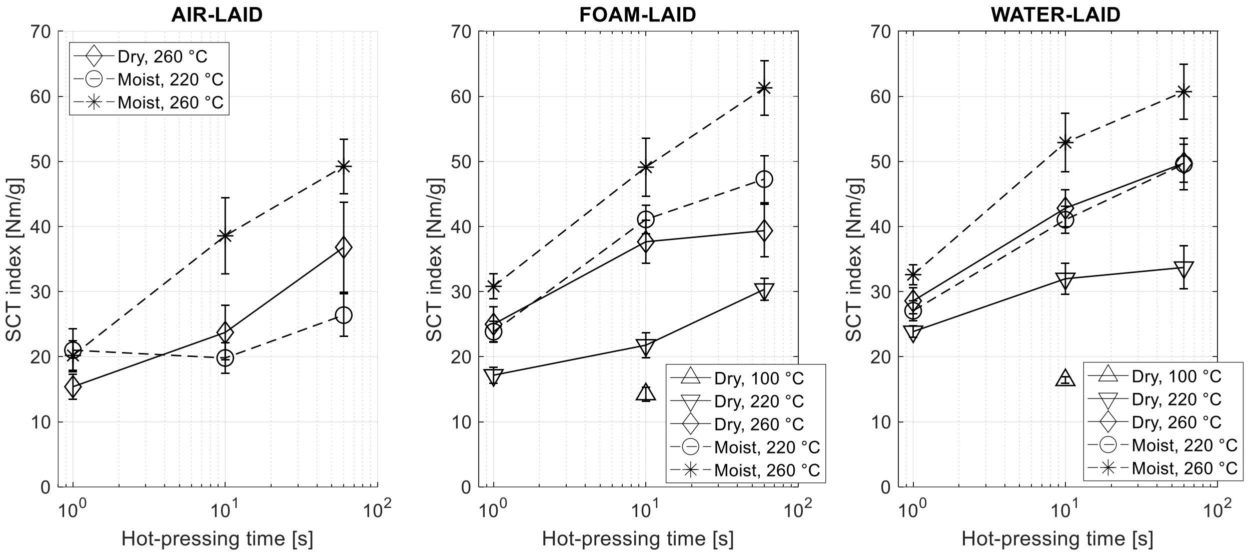

Figure S16. SCT index. Unpressed references: FL (12.0 ± 0.7) Nm/g, WL (14.4 ± 0.8) Nm/g.

References

[1] S. Willför *et al.*, "Carbohydrate analysis of plant materials with uronic acid-containing polysaccharides-A comparison between different hydrolysis and subsequent chromatographic analytical techniques," (in English), *Ind Crop Prod,* vol. 29, no. 2-3, pp. 571-580, Mar 2009, doi: 10.1016/j.indcrop.2008.11.003.

[2] O. Goldschmid, "Ultraviolet spectra," in *Lignins: Occurrence, formation, structure and reactions*, K. V. Sarkanen and C. H. Ludwig Eds. New York: John Wiley & Sons, 1971, pp. 241-298.

[3] S. Suuronen, "Effect of autohydrolysis on the kinetics of alkaline cooking," Master of science, Faculty of chemistry and materials sciences, Aalto university, 99, 2010.

[4] A. Mattsson, T. Joelsson, A. Miettinen, J. A. Ketoja, G. Pettersson, and P. Engstrand, "Lignin Inter-Diffusion Underlying Improved Mechanical Performance of Hot-Pressed Paper Webs," (in English), *Polymers-Basel,* vol. 13, no. 15, Aug 2021, doi: 10.3390/polym13152485.
